# Sequence-specific DNA binding by MYC/MAX to low-affinity non-E-box motifs

**DOI:** 10.1371/journal.pone.0180147

**Published:** 2017-07-18

**Authors:** Michael Allevato, Eugene Bolotin, Mark Grossman, Daniel Mane-Padros, Frances M. Sladek, Ernest Martinez

**Affiliations:** 1 Department of Biochemistry, University of California Riverside, Riverside, California, United States of America; 2 Department of Cell Biology and Neuroscience, University of California Riverside, Riverside, California, United States of America; University of Manchester, UNITED KINGDOM

## Abstract

The MYC oncoprotein regulates transcription of a large fraction of the genome as an obligatory heterodimer with the transcription factor MAX. The MYC:MAX heterodimer and MAX:MAX homodimer (hereafter MYC/MAX) bind Enhancer box (E-box) DNA elements (CANNTG) and have the greatest affinity for the canonical MYC E-box (CME) CACGTG. However, MYC:MAX also recognizes E-box variants and was reported to bind DNA in a “non-specific” fashion *in vitro* and *in vivo*. Here, in order to identify potential additional non-canonical binding sites for MYC/MAX, we employed high throughput *in vitro* protein-binding microarrays, along with electrophoretic mobility-shift assays and bioinformatic analyses of MYC-bound genomic loci *in vivo*. We identified all hexameric motifs preferentially bound by MYC/MAX *in vitro*, which include the low-affinity non-E-box sequence AACGTT, and found that the vast majority (87%) of MYC-bound genomic sites in a human B cell line contain at least one of the top 21 motifs bound by MYC:MAX *in vitro*. We further show that high MYC/MAX concentrations are needed for specific binding to the low-affinity sequence AACGTT *in vitro* and that elevated MYC levels *in vivo* more markedly increase the occupancy of AACGTT sites relative to CME sites, especially at distal intergenic and intragenic loci. Hence, MYC binds diverse DNA motifs with a broad range of affinities in a sequence-specific and dose-dependent manner, suggesting that MYC overexpression has more selective effects on the tumor transcriptome than previously thought.

## Introduction

The oncoprotein MYC is an essential DNA-binding transcription factor of the basic helix-loop-helix leucine zipper (bHLHZ) family that regulates transcription of a large number of genes in metazoans and controls cell cycle, cell growth, metabolism, cell proliferation, differentiation, apoptosis, and cell transformation and is overexpressed in many types of cancer [1, 2]. MYC binds DNA as a heterodimer with an obligatory bHLHZ partner called MAX. In contrast to MYC, the MAX protein can also homodimerize. The MYC:MAX heterodimer and MAX:MAX homodimer recognize Enhancer box (E-box) DNA elements (CANNTG) and have the highest affinity for the palindromic E-box sequence CACGTG, referred to hereafter as the canonical MYC E-box (CME) [3–9]. Heterodimerization with MAX is essential for most MYC biological functions, including transcription regulation and cell transformation [3, 6, 10, 11]. In contrast MAX:MAX homodimers do not have a transcription regulatory domain and interfere with MYC:MAX functions via recognition of the same E-box motifs [12, 13].

Crystal structures of the bHLHZ domains of MYC:MAX and MAX:MAX complexes (referred to as MYC/MAX complexes hereafter) bound to a CME-containing oligonucleotide showed that three conserved amino acids within the basic region of MYC and MAX make identical contacts with four specific bases located symmetrically on either arm of the palindromic CME sequence CACGTG (base contacts underlined for only one strand) [14, 15]. *In vitro* binding site selection studies showed that MYC/MAX complexes can also bind non-canonical motifs, albeit with reduced affinities, including the E-box sequence CATGTG and several E-box variants such as CACGCG, CACGAG, CATGCG, and CACGTT (non canonical base underlined) [5, 9].

Genome-wide chromatin immunoprecipitation followed by high throughput sequencing (ChIP-seq) in mammalian cells confirmed that the CME (CACGTG) is the most significantly enriched sequence at MYC-bound loci *in vivo* followed by the variant sequences CACGCG, CATGCG, CACGAG, and CATGTG previously identified *in vitro* [16–19]. Both *in vitro* and *in vivo* studies also indicated that MYC/MAX affinity for core E-box elements is influenced by nucleotides immediately flanking the core hexamer [5, 17]. ChIP-seq analyses further revealed that MYC often co-localizes with CpG islands [19, 20]; in the genome of mouse fibroblasts, for example, about half of MYC-bound sites occur within 1 kb of a transcription start site (TSS), a majority (~75%) of which are CpG islands [17]. The genomic occupancy of MYC:MAX has also been shown to correlate with an active/open chromatin conformation [21, 22] and with the occupancy of the RNA polymerase II (RNAPII) machinery [23]. This is consistent with not only the known interactions between MYC and the RNAPII machinery, other DNA-binding regulators and chromatin-modifying coregulators [1, 24–26], but also with the observation that MYC stimulates the release of pre-engaged and paused RNAPII at many promoters in embryonic stem cells [27]. More recently, it was noted that under certain *in vitro* conditions MYC:MAX complexes display non-specific DNA-binding activity and that MYC binding to chromatin *in vivo* does not generally correlate with the presence of high affinity motifs, including the top 12 motifs identified *in vitro* [23]. This led to the proposal that MYC genomic occupancy is largely independent of sequence specificity and that MYC:MAX complexes are primarily recruited by other chromatin-associated factors and/or epigenetic marks [23, 25, 28].

In this study, in order to address the issue of nonspecific DNA binding by MYC/MAX, we used recombinant MYC/MAX complexes in a protein-binding microarray (PBM) approach with which we could examine binding to 5000 unique DNA sequences, without interference of other host factors. We show that MYC/MAX complexes bind in a sequence-specific manner to not only high-affinity E-boxes and close variants but also to more degenerate and lower affinity non-E-box DNA sequences, such as the palindromic hexamer AACGTT, which we confirmed by electrophoretic mobility-shift assays (EMSAs). Bioinformatic analyses of published ChIP-seq datasets indicated that the vast majority (87%) of MYC-occupied genomic loci in human cells contained at least one of the top 21 MYC-bound motifs identified in the PBM, including the low-affinity AACGTT hexamer. Furthermore, we found differential effects of MYC expression levels on binding to high-affinity CME versus low-affinity non-E-box AACGTT motifs as well as promoter versus distal regions. In addition, the top biological processes preferentially regulated via MYC-bound CME genes (e.g., ribosome biogenesis, metabolic processes) are not enriched for in genes with MYC-associated AACGTT motifs. Hence, we propose that low-affinity binding sites could play an important role in conveying specific MYC functions under conditions of overexpression, which occur during transformation to a cancerous state.

## Materials and methods

### Recombinant MYC/MAX complexes

Recombinant six histidine (6his)-tagged full-length human MYC (p64; UniProtKB P01106-1) and MAX (p21; UniProtKB P61244-2) proteins were expressed in *E*. *coli*, purified and reconstituted to form functional MYC:MAX heterodimeric complexes, essentially as previously described [29], with the exception that RosettaBlue(DE3)pLacI cells were used to achieve higher expression of recombinant 6his-MYC. MYC:MAX complexes were verified to be devoid of MAX:MAX dimers by electrophoretic mobility shift assay (EMSA).

### MYC/MAX protein binding microarray (PBM)

**Fig 1:**
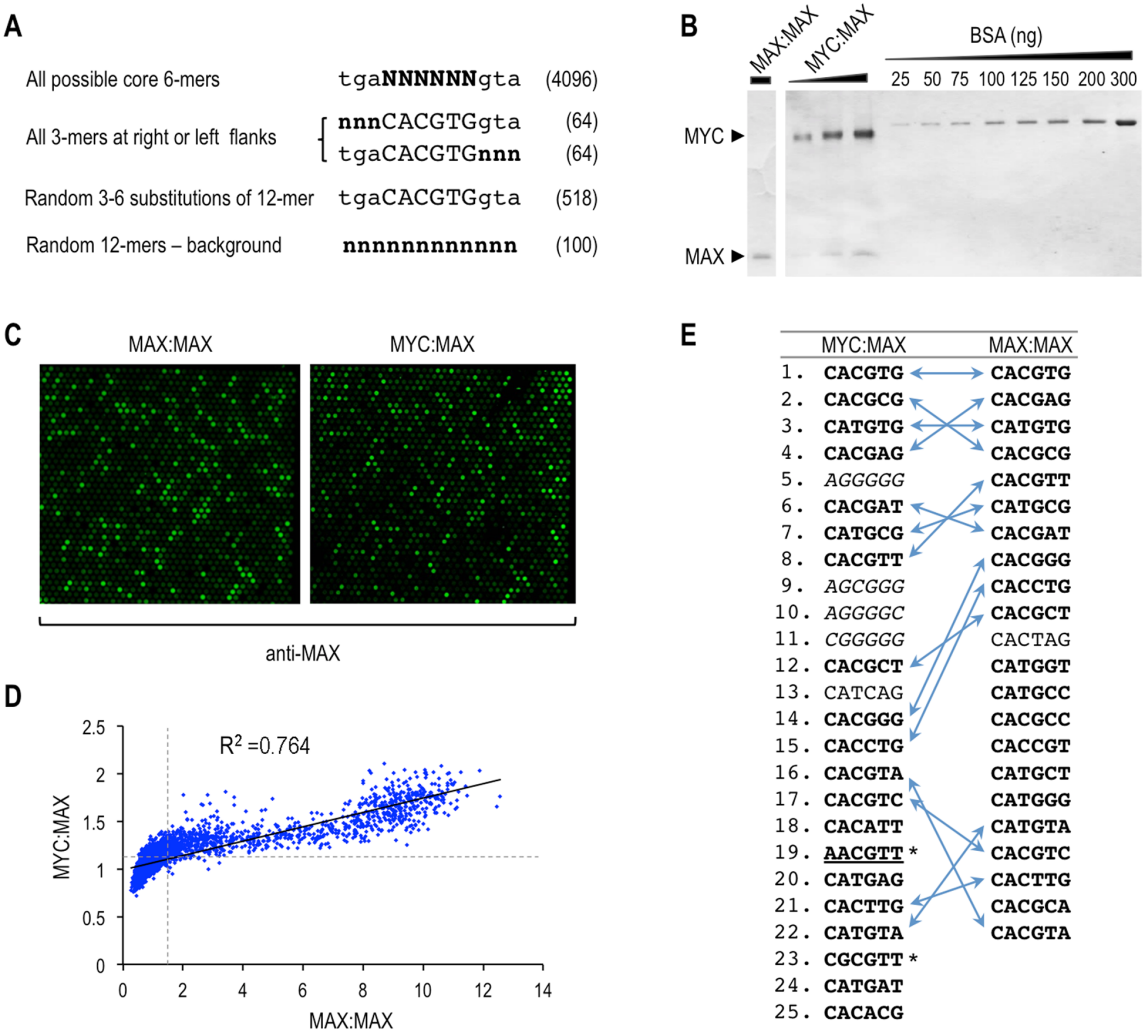
PBM analysis of the DNA-binding specificity of MYC/MAX complexes. **(A)** The custom PBM design contained immobilized DNA probes in triplicates with the indicated sequences (N and n indicate the varying positions having any of the 4 nucleotides). The total number of different sequences is shown in brackets. **(B)** Coomassie-stained SDS-PAGE showing the purified recombinant MYC/MAX complexes. **(C)** Images of fluorescent spot signals on PBM slides bound by MYC:MAX and MAX:MAX complexes and probed with anti-MAX antibody. **(D)** Scatter plot illustrating the correlation between MYC:MAX and MAX:MAX binding scores (normalized fluorescent intensities) for each individual probe spotted on the PBM (R^2^ = 0.764). Dashed lines indicate the threshold values for binding scores above 80% of random probes. **(E)** Ranked order list of top-scoring motifs in PBM probes bound by MYC:MAX and MAX:MAX (with binding scores above 95% of random probes). Double arrows indicate identical motifs. Itallics are the low complexity G/C-rich motifs. Asterisks indicate non-E-box motifs, including the AACGTT motif (underlined). Motifs highlighted in bold were bound by both MYC:MAX and MAX:MAX above the 80% random threshold.

Protein binding microarrays (PBMs) were carried out essentially as previously described [30] except that purified recombinant MYC/MAX proteins complexes were used. A custom-designed array was ordered from Agilent and contained 15,000 spots of single-stranded DNA probes with ~5000 unique DNA sequences each spotted in triplicate as 12-nucleotide test sequences and attached to a glass slide (designed as in Fig 1A). The variable 12-nucleotide sequence was located between the linker (5’- CGTCGATATAGTAATCTTAGCTATTAA-3’) and the cap (5’-GCCGG-3’). All the unique DNA sequences of the array are provided in supplemental S1 Table. The DNA probes were made into double-stranded duplexes using a primer to a common linker sequence, dNTPs (GE Healthcare) and Thermo Sequenase (Affymetrix, Cat# 78500). The slides were subsequently blocked for 90 min with 2% non-fat milk in PBS plus 0.01% Tween 20, washed three times (all washes were done with PBS plus 0.01% Tween 20 unless otherwise specified) and then incubated with 550 ng MAX:MAX or MYC:MAX complexes in binding buffer (15 mM Tris-HCl pH 7.5, 100 mM KCl, 0.075% NP40, 7.5 mM beta-mercatoethanol, 375 ng/μl BSA and 8 ng/μl poly(dA–dT):(dA–dT) for 60 min. After three washes the slides were incubated overnight with a primary antibody to MAX diluted 1:200 (rabbit anti-MAX C-17X, Santa Cruz), washed three times and then incubated with donkey anti-rabbit Dylight 549 (Jackson ImmunResearch) at 1:50 dilution for 60 min. Primary and secondary antibodies were diluted in 2% milk plus 0.01% Tween20. All reactions were carried out at room temperature. After washing three times, plus a final wash in PBS, the slide was dried and scanned at 543 nm using a GenePix Axon 4000B scanner (Molecular Devices, Sunnyvale, CA) at the UCR Genomics Core Facility. Extraction and normalization of the data were as described previously [30]. MEME (meme-suite.org) was used for unbiased motif discovery and generation of position weight matrices as sequence logos [31]. PBM data are publicly available on the Nuclear Receptor DNA Binding website (http://nrdbs.ucr.edu), Accession numbers PBM7.1MAX09072010 and PBM7.1MYCMAX09072010.

### Electrophoretic mobility-shift assay (EMSA)

Complementary pairs of oligonucleotides consisting of the Consensus MYC E-box (CME), Non-E-box (NE), derivative sequence variants, and Control (Ctrl.) sequences were purchased from Eurofins Genomics, annealed and labeled by Klenow filling with [α-32P]dGTP to a specific activity of ~4 × 10^5^ cpm/pmol. DNA-binding with recombinant MYC/MAX complexes were performed in 10-μl reactions containing 0.5 pmol labeled probe, 80 ng poly(dA–dT):(dA–dT), 375 ng/μl BSA, 15 mM Tris–HCl (pH 7.9), 100 mM KCl, 15% glycerol, 0.15 mM EDTA, 0.075% NP-40, and 7.5 mM 2-mercaptoethanol, essentially as previously described [29]. DNA-binding competition assays were performed similarly, but unlabeled competitor oligos were mixed with the radiolabeled CME probe before the proteins were added. DNA-binding reactions were analyzed on a 6% native polyacrylamide gel, as described [29]. After drying the gel was autoradiographed and signals were quantified with a PhosphoImager (Typhoon 9410; GE Healthcare). The fraction (%) of bound probe remaining (Bound) is relative to no competitor and is the mean ± S.D. of more than three independent experiments. Statistical significance was determined by two-tailed Student’s t-test and P<0.05.

### Bioinformatics analyses

#### Datasets, filtering, and quality assessment

MYC-bound genomic sequences in the MYC-inducible human B cell line P493-6 were obtained from Gene Expression Omnibus (GEO) ChIP-seq reads derived from cells under either “high” (GSM1234501) or “low” (GSM1234500) levels of MYC [22]. GEO sample GSM894093 was used as the input control. ChIP-seq data sets of MYC-bound sequences in U2OS cells were obtained from GEO: GSE77356 [28]. SRA files were converted to FASTQ via NCBI SRA toolkit and uploaded to the public Galaxy server (http://usegalaxy.org) [32–34]. ChIP-seq reads were filtered using FASTQ Groomer and evaluated using the FASTQC tool.

#### Analysis of ChIP-seq data

For peak finding, ChIP-seq reads were aligned to the UCSC human genome (hg19) using Bowtie for Illumina. Unmapped reads were removed and the SAM files were converted to BAM files. The BAM files were transferred to a Galaxy server dedicated to ChIP-seq analysis, Cistrome (http://cistrome.org) [35]. MYC peaks were called using the MACS software version 2. Only significant peaks with P values below 1e-08 were recognized. MACs peaks were ranked by fold enrichment and sorted into three subsets: “Low”, “Medium” and “High” enriched regions. The apex of each MYC peak was expanded by +/-100 base pairs to obtain MYC summits.

For motif enrichment and annotation, the genomic locations of all E-boxes, the Non-E-box (AACGTT) and control sequences were determined using the HOMER (http://homer.salk.edu) motif discovery software [36]. The normalized frequencies of motifs in MYC peaks and summits were obtained by intersecting the genomic positions of ChIP-seq peaks/summits and motifs, and then normalizing to the occurrence of each motif in the genome. Homer’s peak annotation program was used to associate MYC peaks and summits with nearby genes and to identify peaks in the promoter (+/- 2 kb of TSS), intergenic, or intragenic regions of a genomic feature. Homer’s peak annotation tool was also used to determine the spatial distribution of the CME and NE motifs in MYC summits (+/-100bp) and summits extended by 250bp on either side (total region +/-350bp).

For the gene ontology analyses, the genes associated with MYC peaks or summits containing only CME or NE motifs were analyzed by using Metascape (http://metascape.org) [37]. The annotation field “Biological Process” was used in the analyses.

## Results

### Identification of DNA sequence motifs preferentially bound by MYC/MAX complexes *in vitro*

In order to identify in an unbiased manner the DNA sequences preferentially bound by MYC/MAX complexes, we designed a protein binding microarray (PBM) that contained ~5000 different DNA probes (all in triplicate) and included all possible 6-mer motifs (tgaNNNNNNgta, 4096 total; Fig 1A). In addition, the PBM contained 128 probes with all 3-mer substitutions in either the left or the right flank of the CME core (nnnCACGTGgta and tgaCACGTGnnn) and 518 random substitutions of the 12-mer CME motif tgaCACGTGgta (minimum of three and maximum of six substitutions within a given 12-mer sequence). The PBM also contained 100 random 12-mer probes, which were used to evaluate non-specific binding (see Fig 1A). The PBM was incubated with purified recombinant full-length MYC:MAX or MAX:MAX protein complexes (Fig 1B) and analyzed by immunofluorescence using an anti-MAX antibody (Fig 1C). The MYC:MAX complexes were devoid of any MAX:MAX dimer as determined by EMSA (see below).

Global analysis of the ~5000 DNA sequences on the PBM indicated, as expected, that the binding sequence preferences of MYC:MAX strongly correlate to those of MAX:MAX (R^2^ = 0.764, Fig 1D). When considering only the most significantly bound probes in the PBM (binding scores greater than 95% of the random 12-mer sequences, corresponding to >2 standard deviations from mean random score), we identified 378 probes with 25 different motifs bound by MYC:MAX and 466 probes with 22 different motifs bound by MAX:MAX (Fig 1E, S1 Table). Among these, a total of 14 different 6-mer motifs were common to both MYC:MAX and MAX:MAX (Fig 1E, double arrows). The top six common MYC/MAX motifs matched the top six motifs identified by Guo et al. [23] in an independent PBM analysis reported while our study was in progress (see S1 Table). Two other MYC-bound motifs identified by Guo et al. in their top-12 list (Guo’s motifs #7 and #10) were also in our list of top binders (see S1 Table). As expected, the CME motif CACGTG had the highest binding score for both MYC:MAX and MAX:MAX (Fig 1E). Nearly all the remaining top-ranked MYC/MAX motifs were either E-boxes (CANNTG) or variants having at least one half E-box sequence of the type CAC or CAT (Fig 1E). Notable exceptions were the motifs AACGTT and CGCGTT (asterisks in Fig 1E). These motifs diverge from typical CANNTG E-boxes (and from the CME) by two substitutions in both the CA and TG arms that define the palindromic E-box (underlined above), and hence were referred to as “non-E-box” sequences hereafter. In addition, the low complexity G/C-rich motifs AGGGGG, AGCGGG, AGGGGC, and CGGGGG (italics in Fig 1E) were also found in top-ranked probes bound by MYC:MAX. However, these motifs were not bound by MAX:MAX and, therefore, were not investigated further (see Discussion). Using a less stringent binding threshold (binding scores greater than 80% of random sequences) the non-E-box palindromic sequence AACGTT and all other motifs indicated in bold in Fig 1E (a total of 27 top-ranked motifs) were bound by both MYC:MAX and MAX:MAX. These represent high-confidence MYC/MAX motifs.

**Fig 2:**
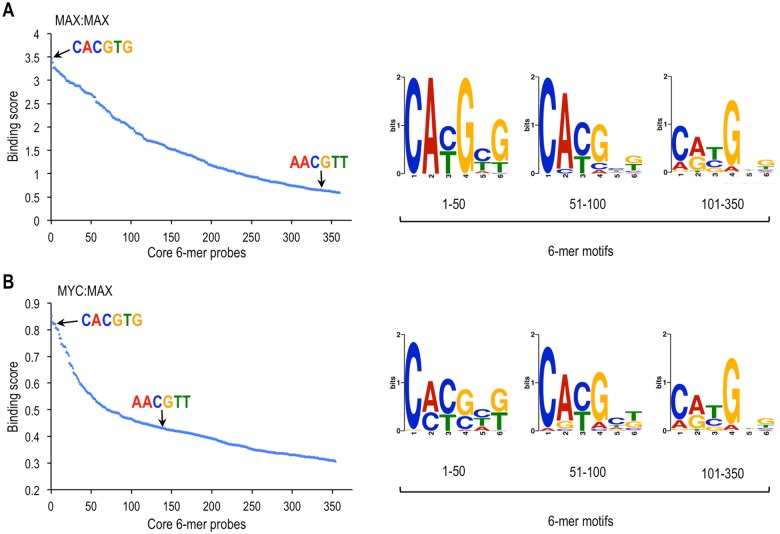
Position weight matrices of ranked core 6-mers bound by MAX:MAX and MYC:MAX. Core 6-mer probes of the PBM were ranked according to their binding scores (log2 of normalized fluorescent intensities) for MAX:MAX **(A)** and MYC:MAX **(B)**. Only probes scoring above 80% of random sequences are shown. The highest scoring probes containing the CME (CACGTG) and the NE (AACGTT) are indicated with arrows. Position weight matrices of 6-mer motifs present in the top 1–50, 51–100, and 101–350 bound probes were obtained with MEME and are represented as logos. The 8-mer sequences of bound probes (i.e., aNNNNNNg) were used as input for motif discovery.

We further selectively analyzed the 4096 probes having every 6-mer motif flanked by identical nucleotides (i.e., tgaNNNNNNgta) and found ~360 probes specifically bound by MAX:MAX (Fig 2A) and MYC:MAX (Fig 2B) (with scores higher than 80% of random sequences). These probes contained most of the top 6-mer motifs for MYC:MAX and MAX:MAX identified in Fig 1E (see also S1 Table), and included the 27 motifs bound by both MYC:MAX and MAX:MAX (shown in bold in Fig 1E). As expected, the motifs of the top 50 bound probes were generally close variants of the canonical CME sequence CACGTG for both MAX:MAX and MYC:MAX (Fig 2A and 2B; logos 1–50). MYC:MAX also bound the low complexity G/C-rich sequence CCCCCT (or AGGGGG) and variants thereof (Fig 2B; logos 1–50). The next 50 probes with intermediate scores (Fig 2A and 2B; logos 51–100) had more degenerate motifs but often retained one half E-box and a central CG or TG (e.g., CACGNN or CATGNN). The remaining 150 probes with the lowest binding scores (Fig 2A and 2B, logos 101–350) had highly degenerate motifs that often had the first C replaced by A, but maintained the central CG or TG dinucleotides (e.g., CACGNN, CATGNN, or AACGNN). Notably, this analysis also identified the palindromic sequence AACGTT as a new non-E-box motif bound by both MYC:MAX and MAX:MAX complexes (Fig 2A and 2B, left panels).

The PBM data further indicated that in the context of the CME motif CACGTG there was a moderate influence of the flanking nucleotides, with possible differential effects on MYC:MAX and MAX:MAX (S1–S4 Figs). For instance, the top 25% of CME motifs most avidly bound by MAX:MAX were enriched in flanking sequences with a T or G at position n^1^ of the 12-mer n^1^n^2^n^3^CACGTGgta, while MYC:MAX-bound motifs often had an A at position n^3^ (S1 and S3 Figs). However, both MYC:MAX and MAX:MAX complexes bound poorly to sequences with an A at position n^10^ of the 12-mer tgaCACGTGn^10^n^11^n^12^, as those sequences were most often found in the group of bound probes with the lowest binding scores (bottom 25%, S2 Fig). Further analysis of bound probes having three to six random substitutions of the 12-mer tgaCACGTGgta also suggested distinct sequence preferences between MYC:MAX and MAX:MAX (S4 Fig). The significance of this differential binding is as of yet unclear, but merits further investigation. The complete ranked lists of the PBM probe sequences and binding scores for MYC:MAX and MAX:MAX are provided as supplemental information (S1 Table).

### Sequence-specific binding of MYC/MAX to the non-E-box motif AACGTT

**Fig 3:**
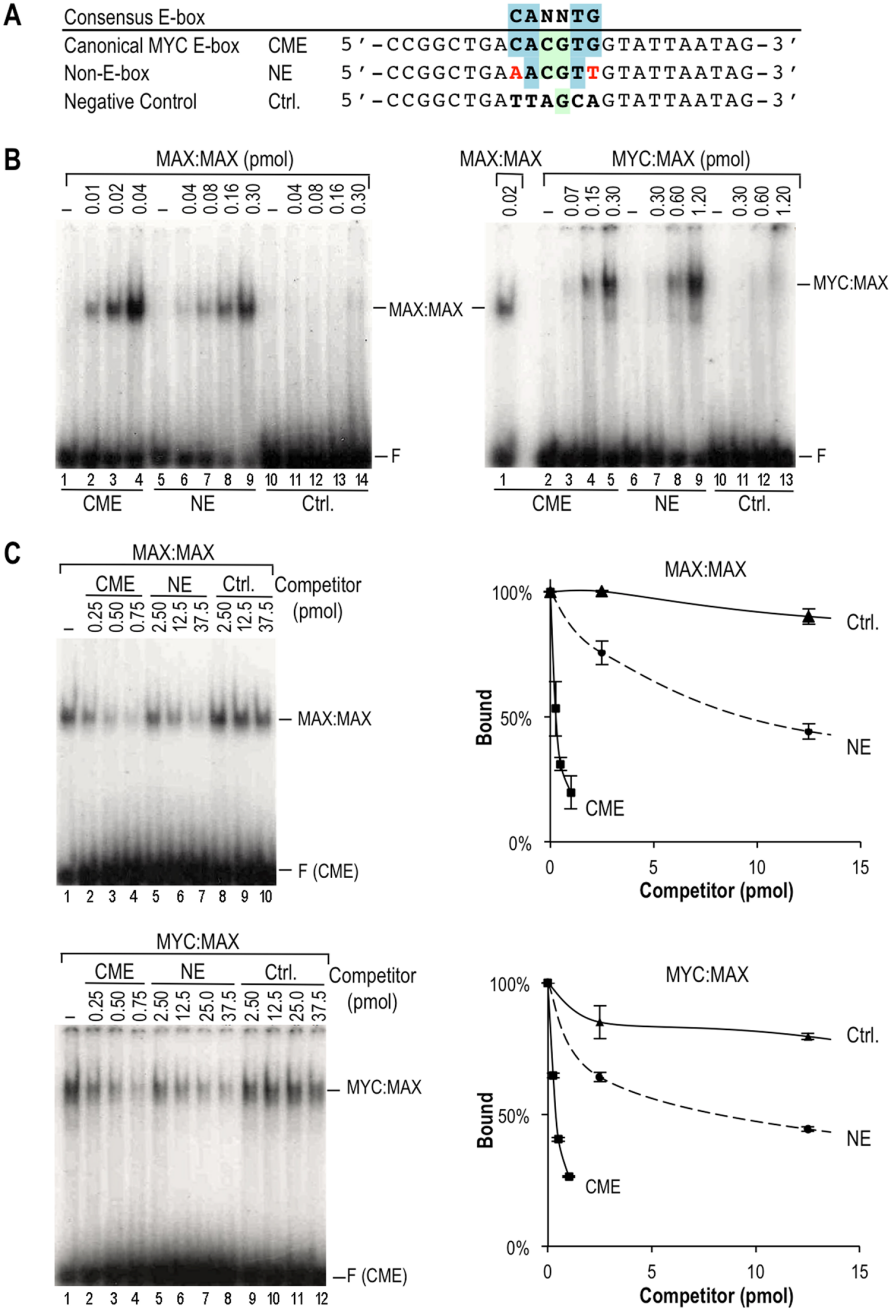
Specific binding of MYC/MAX complexes to the non-E-box (NE) motif AACGTT. **(A)** Sequences of the probes used in EMSA. Nucleotides in red indicate positions in the NE probe that deviate from an E-box. **(B)** EMSA with the CME, NE and Ctrl probes and with the indicated amounts (pmol) of MAX:MAX and MYC:MAX. The position of the unbound/free probe is shown (F). **(C)** EMSA competition assays were performed with the labeled CME probe and the indicated MYC/MAX complex and the indicated amounts (pmol) of either CME, NE or Ctrl competitor oligos or no competitor (-). Two representative gels are shown (left). Results from three independent replicates were quantitated and the means (± S.D.) were plotted (right) and significantly differed (p<0.05) between the competitor oligos.

Recognition of the canonical MYC E-box sequence (CME) by MYC/MAX complexes involves important symmetrical contacts with specific bases (underlined for only the top strand CACGTG), including at the first and last positions of the core hexamer [14, 15]. Consequently, it was intriguing to find that the non-E-box (NE) palindromic sequence AACGTT, which has substitutions at both the first and last positions, was specifically bound by MYC/MAX in the PBM. To further verify the specificity of this interaction we used EMSA to analyze the binding of MYC:MAX and MAX:MAX complexes to radiolabeled CME, NE and non-specific DNA (Ctrl) probes (Fig 3A). Both MYC:MAX and MAX:MAX complexes bound specifically to the CME and NE probes (no binding to the Ctrl probe was observed) but the binding to the NE probe required higher protein concentrations, indicative of a lower affinity (Fig 3B). To measure the relative affinities of MYC:MAX and MAX:MAX for the CME and NE motifs we performed EMSA competition experiments in which different amounts of cold CME, NE or Ctrl oligonucleotides were co-incubated with the labeled NE probe and MYC/MAX complexes (Fig 3C). Compared to the CME, the NE motif had a significantly lower affinity for MYC:MAX and MAX:MAX (about 20- and 33-fold lower, respectively), while the Ctrl oligo did not compete, as anticipated (Fig 3C).

**Fig 4:**
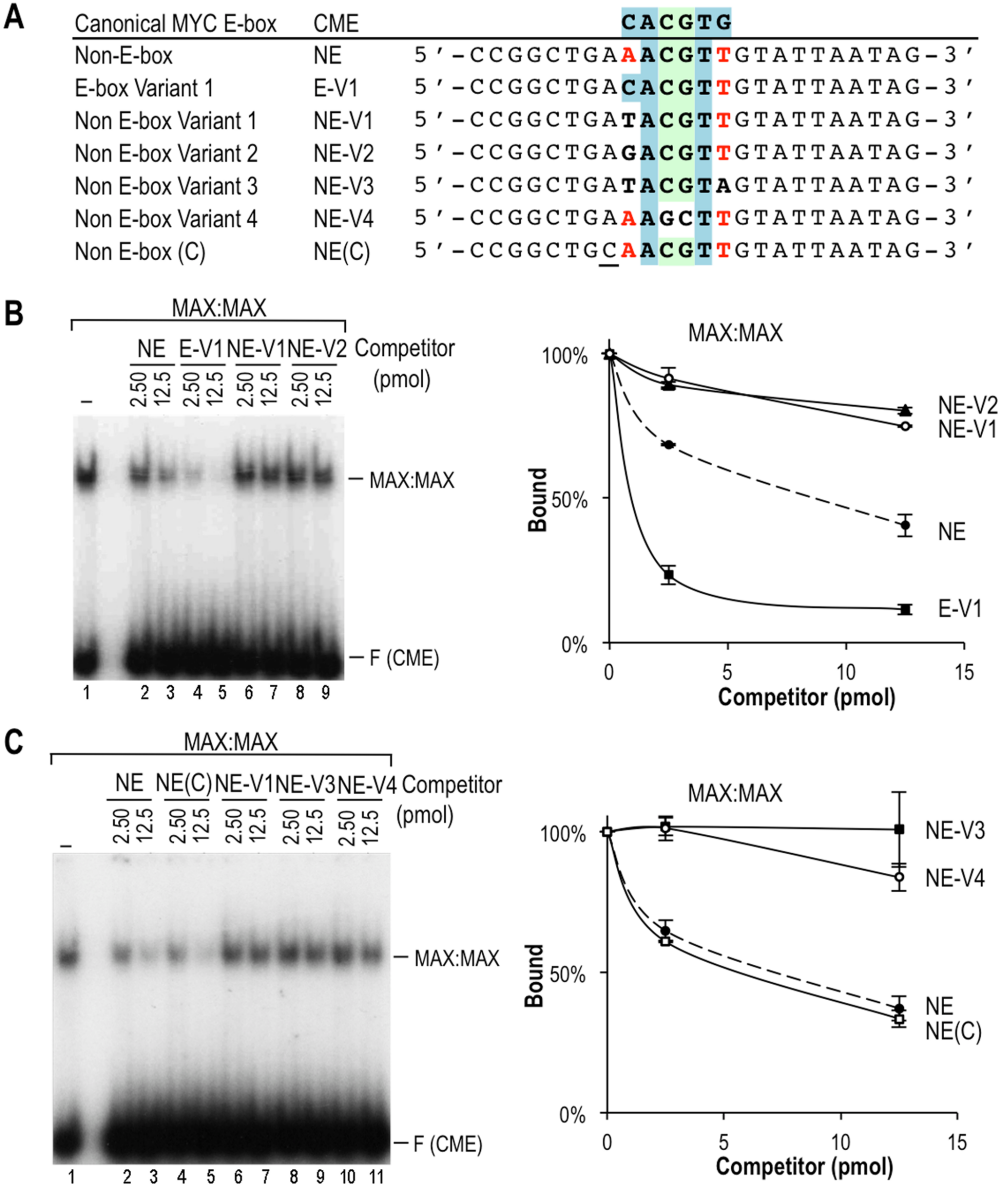
Sequence requirements for MYC/MAX binding to the NE motif AACGTT. **(A)** Sequences of the oligo competitors. **(B, C)** EMSA competition assays with MAX:MAX and the labeled CME probe and the indicated amounts (pmol) of competitor oligos. Three independent experiments were quantitated as in Fig 3C. Significant differences were observed between NE and NE-V1/-V2/V3/V4 (p<0.005 by two-tailed t-test).

**Fig 5:**
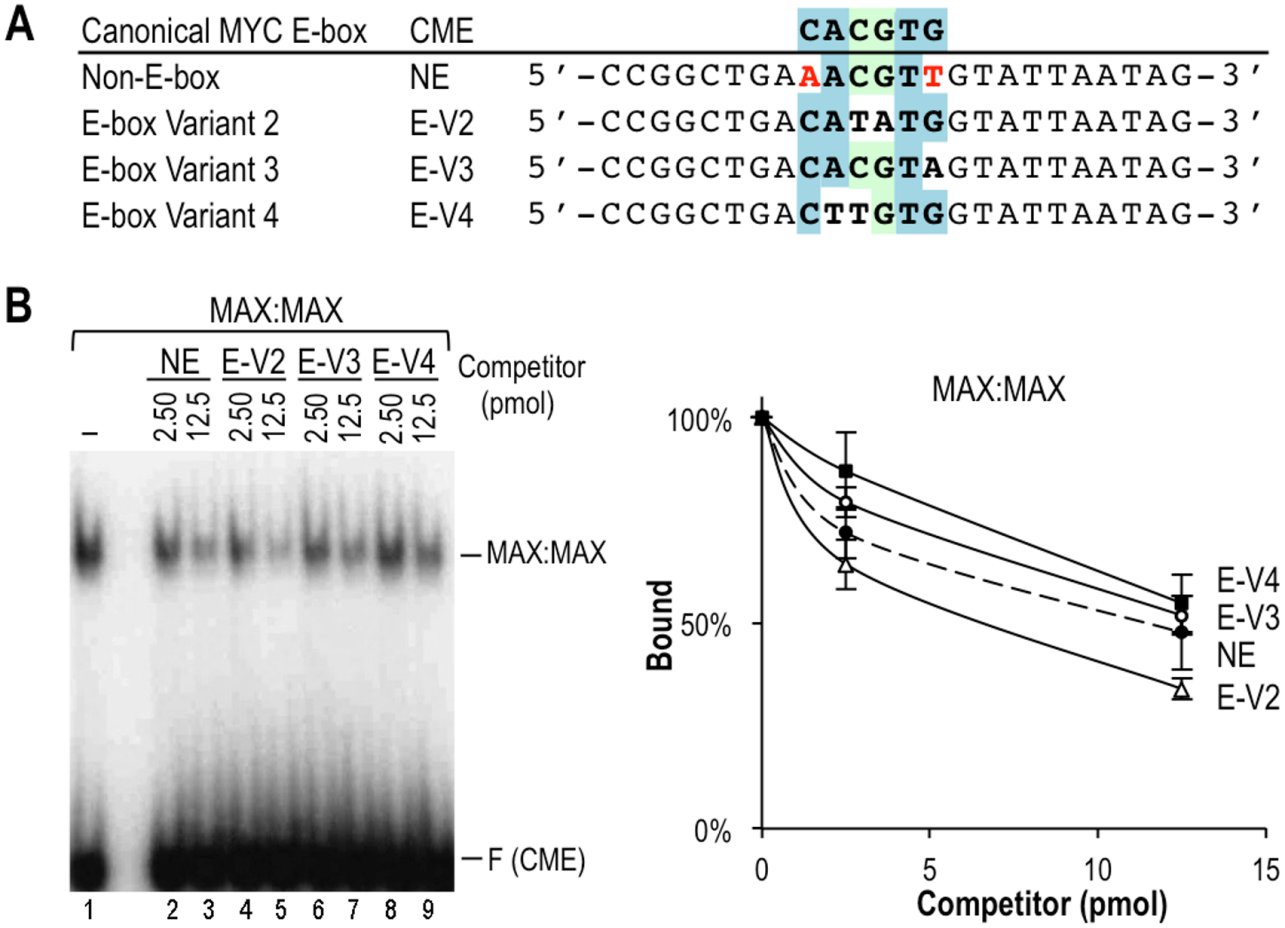
The NE motif AACGTT and several E-box variants have a similar affinity for MAX:MAX. **(A)** Sequences of the oligo competitors. **(B)** EMSA competition assays with MAX:MAX and the labeled CME probe and the indicated amounts (pmol) of competitor oligos. Three independent experiments were quantitated as in Fig 3C. No statistically significant difference was observed between NE and the different competitors (p>0.05 by two-tailed t-test).

**Fig 6:**
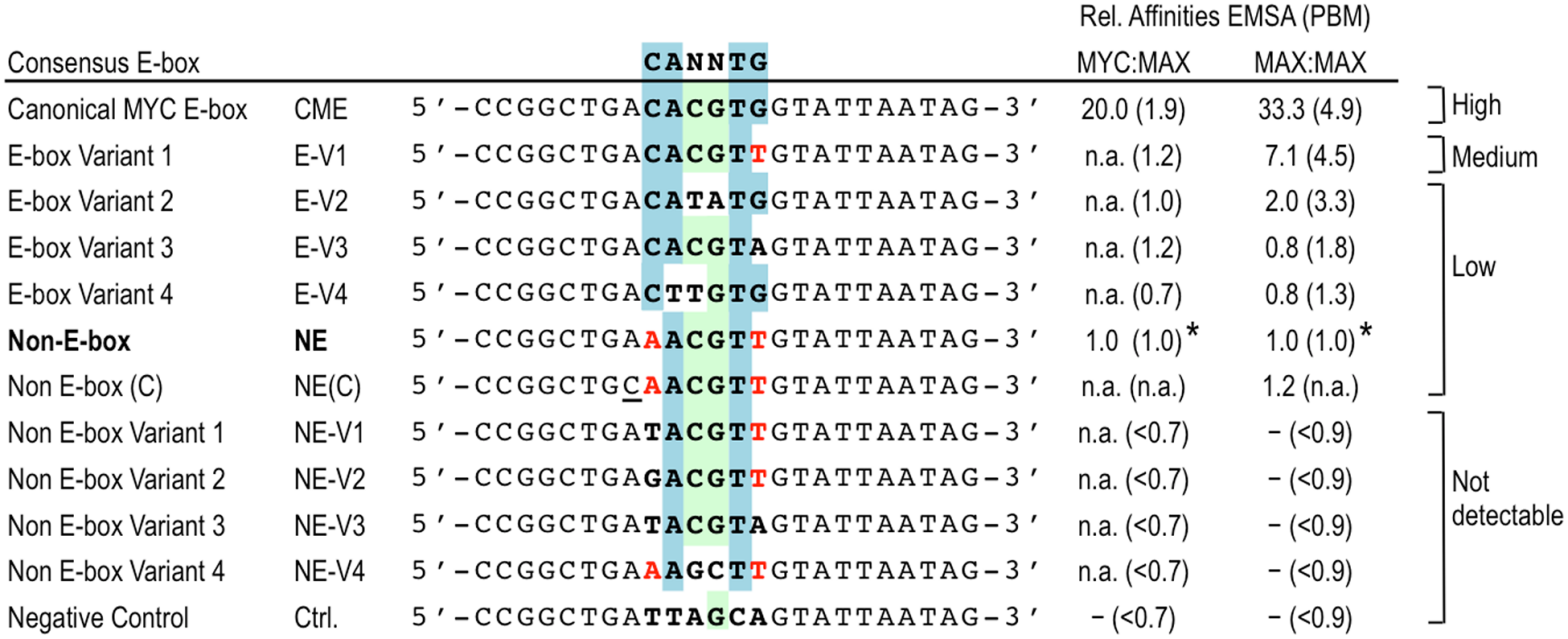
Summary of relative affinities of MYC:MAX and MAX:MAX complexes for different E-box and non-E-box sequences. Affinities were normalized relative to the affinity of the NE sequence, which was arbitrarily set to 1 (*). Relative affinities from the PBM scores are indicated between brackets. Sequences not analyzed by EMSA are indicated by “n.a.” and non-detectable binding is indicated by “-“.

To determine which specific nucleotide positions within the NE motif are important, we performed additional EMSA competition experiments with NE variant (NE-V) sequences and MAX:MAX complexes. Substitution of the first and/or last base pair of the NE sequence AACGTT as in NE variants NE-V1 to V3, or inversion of the central CG nucleotides to GC (i.e., AAGCTT in NE-V4) (Fig 4A) eliminated the competition by the unlabeled oligos indicating that they do not bind MAX:MAX (Fig 4B and 4C). In contrast, a substitution at the first position that restored a perfect half E-box (CACGTT, EV-1) increased competition, and hence binding, and served as a positive control (Fig 4B). Interestingly, a substitution of a flanking nucleotide that generated the palindromic sequence CAACGTTG, which resembles an “extended” CME, did not increase the binding affinity (Fig 4C, NE(C) motif). Finally, the NE had similar binding affinity as several E-box variants that contained one perfect E-box half site CAC/GTG or CAT/ATG (E-V2 to V4, Fig 5) and are known to be specifically bound by MYC:MAX [9]. The relative affinities of the motifs analyzed by EMSA (summarized in Fig 6) were consistent with the binding scores of these sequences in the PBM and identified motifs with “high”, “medium”, and “low” affinity, and several motifs that do not bind MYC/MAX specifically (Fig 6).

### MYC binding to AACGTT-containing genomic loci is highly dependent on increased MYC expression levels

**Fig 7:**
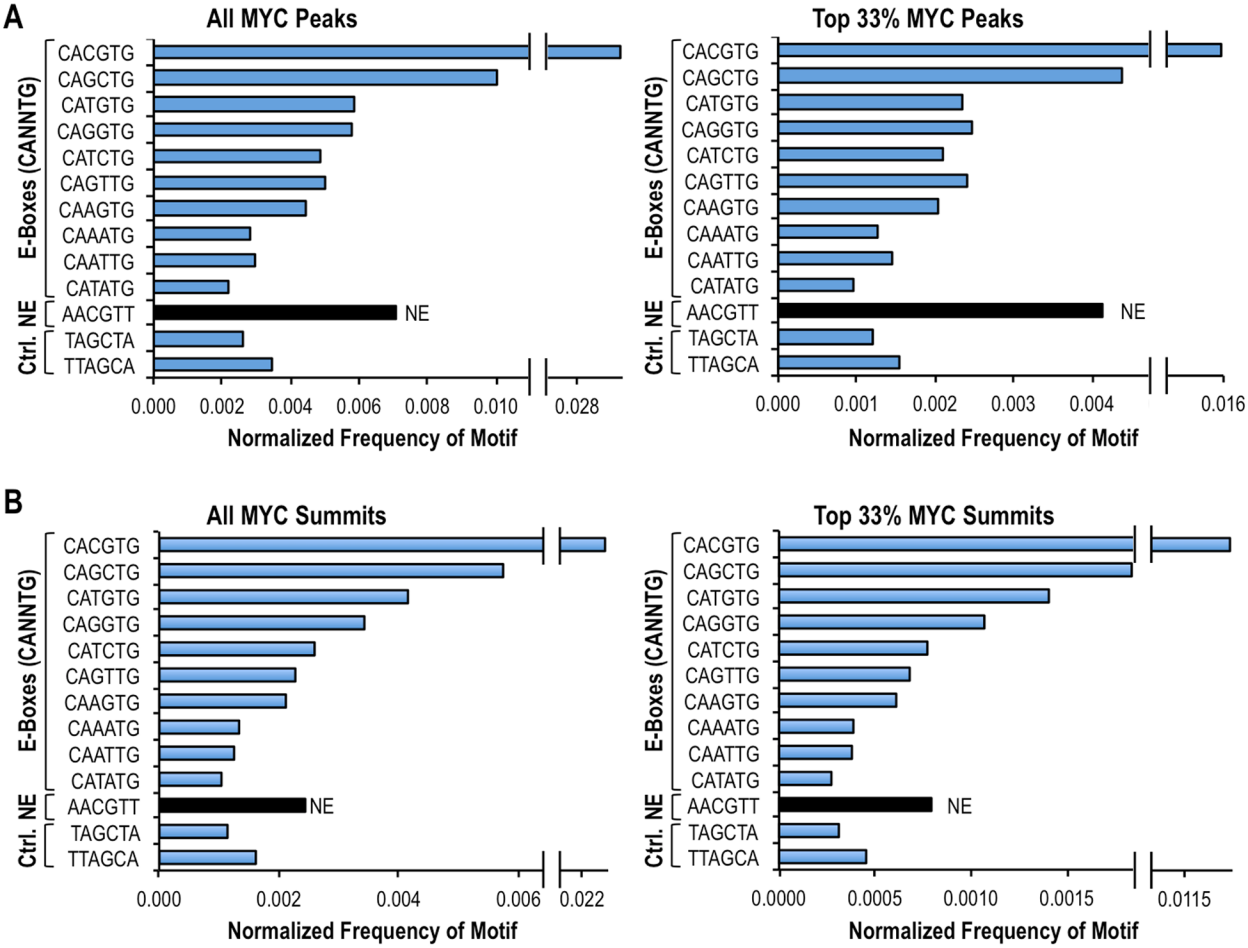
Normalized Frequencies of E-boxes and of the NE motif AACGTT at MYC-occupied genomic loci in human P493-6 B-cells. MYC-bound sequences were obtained from ChIP-seq data of P493-6 cells overexpressing MYC [22]. The frequencies of all ten possible E-boxes (CANNTG), the NE motif AACGTT, and two control (Ctrl) sequences under MYC ChIP-seq peaks **(A)** and summits **(B)** were normalized to the occurrence of each motif in the human genome. The summits are the ±100 bp region from the apex of ChIP-seq peaks. The normalized frequencies are shown for all MYC peaks/summits (left) and for the most significant MYC peaks/summits (top 33% based on p-value; right).

To address whether MYC associates with genomic sites containing the NE sequence AACGTT *in vivo*, we mined previously published MYC ChIP-seq peaks from the human B cell line P493-6 [22]. P493-6 cells carry a tetracycline-repressible ectopic MYC transgene allowing for a range of MYC expression; in the absence of tetracycline the cells express high levels of MYC and display a Burkitt lymphoma-like phenotype [38]. We first compared, in MYC-overexpressing cells, the normalized frequencies (i.e., enrichment) of E-box sequences (CANNTG) and the NE motif AACGTT for all MYC-bound sites (all MYC peaks) or for only the top 33% most significantly bound loci (top 33% MYC peaks, ranked by p-value; Fig 7A). While the CME was the most enriched sequence in both categories, the NE sequence was also enriched under MYC peaks, and was more frequently found than all other E-boxes, with the exception of CAGCTG (Fig 7A). Since MYC ChIP-seq peaks are broad (covering on average ~500 bp), we also examined a more restricted DNA-bound region within 100 bp of the apex (MYC summits), which should contain the primary MYC binding site(s). Within MYC summits, the NE was also more enriched than many E-boxes, but less so than the CME and three other E-boxes, including the sequence CAGCTG (Fig 7B). The latter is intriguing because the CAGCTG sequence was not significantly bound by MYC/MAX in the PBM, consistent with the fact that another class of bHLH proteins (e.g., AP4, E12, E47 and MYOD) recognizes this E-box [39], suggesting an indirect binding of MYC to this element *in vivo*. Notably, the relative *in vitro* binding affinities of MYC/MAX for the specific motifs analyzed by EMSA were generally consistent with the normalized frequencies of these sequences within MYC summits *in vivo* (S5 Fig). Two exceptions were the E-box CATATG, which had a measurable affinity for MYC/MAX *in vitro* but was rarely found in MYC summits *in vivo*; and the NE variant GACGTT (NE-V2), which was not detectably bound *in vitro* but was found at MYC summits *in vivo* (S5 Fig).

**Fig 8:**
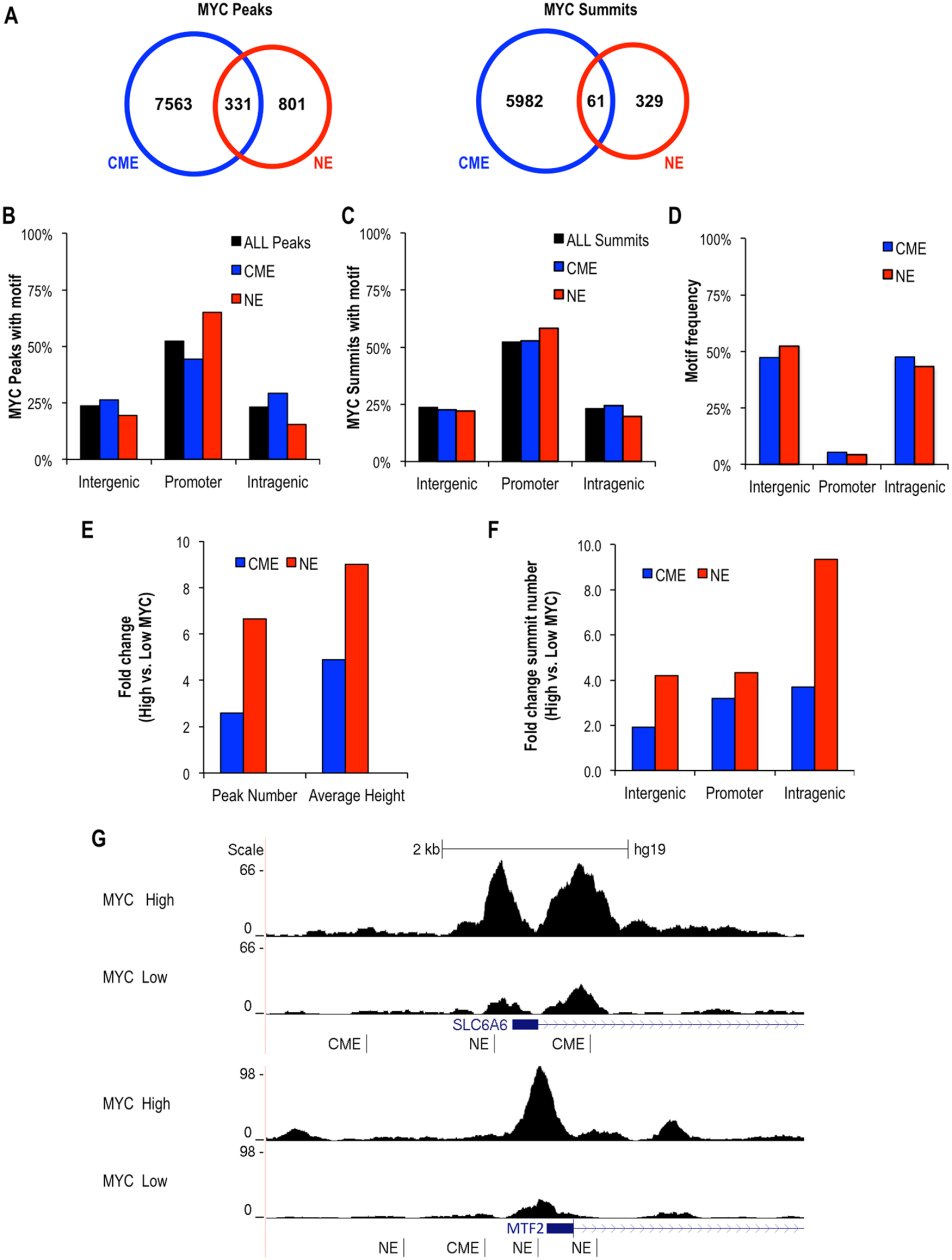
Characterization of MYC-occupied CME (CACGTG) and NE (AACGTT) genomic loci and influence of MYC expression levels in P493-6 cells. **(A)** Venn diagrams show the number of MYC ChIP-seq peaks and summits that contain the CME, the NE or both motifs in high MYC-expressing P493-6 cells. **(B, C)** Frequency distribution plots show the fraction (%) of all MYC ChIP-seq peaks and summits and those specifically containing the CME or NE motifs that are located within promoters (± 2 kb from a TSS), intergenic or intragenic regions. **(D)** Frequency distribution of the CME and NE motifs in the human genome. **(E)** Effect of MYC overexpression on the number and average (mean) height of MYC ChIP-seq summits is shown as fold change (high MYC vs. low MYC) for the summits containing the CME or NE motifs. **(F)** Effect of MYC overexpression on the number of MYC ChIP-seq summits at promoters, intergenic and intragenic regions is shown as fold change (high MYC vs. low MYC) for CME- or NE-containing summits. **(G)** Examples of NE and CME ChIP-seq peaks from human genome browser in P493-6 cells expressing low and high MYC levels.

The above analyses indicate that the NE motif AACGTT is found within MYC-bound sequences *in vivo* at a higher normalized frequency (more enriched) than many E-box motifs. Furthermore, for both the MYC peaks and the more restricted MYC summits the vast majority of MYC-bound sequences with NE motifs (801 out of 1132 and 329 out of 390, respectively) did not contain the CME (Fig 8A). Altogether these results suggest that the NE might be recognized by overexpressed MYC *in vivo*. However, it should be noted that with the exception of the highest affinity and most enriched CME (CACGTG) the other MYC motifs examined, including medium-affinity/medium-enriched motifs (CATGTG and CACGTT) and the low-affinity E-box (CATATG) and NE (AACGTT) did not generally cluster at the center of ChIP-seq summits (S5 Fig). This could suggest that recruitment of MYC to non-CME loci may often involve cooperative interactions with other chromatin-associated factors [21, 23, 25, 28, 40] in addition to sequence-specific binding to low-affinity DNA motifs.

We next characterized the location of different categories of MYC ChIP-seq peaks (all peaks/summits or peaks/summits containing either a CME or NE) relative to annotated genes in MYC-overexpressing cells (Fig 8B and 8C). The NE-containing MYC peaks and summits were mostly (~55–60%) localized to promoter proximal regions (+/-2 kb from a TSS) with the remaining NE-containing MYC peaks/summits equally distributed between intergenic and intragenic locations. A similar distribution was found for all peaks/summits and CME-containing peaks/summits (Fig 8B and 8C). However, this distribution differed remarkably from the global genomic location of CME and NE sites, which were both found primarily in inter- and intragenic regions (Fig 8D), indicating a preferential binding of MYC to both the CME and the NE within promoter regions.

In order to determine whether the cellular concentration of MYC impacts its binding to CME or NE sites *in vivo*, we compared the ChIP-seq peak number and height, as well as position relative to the gene body, in cells expressing high versus low levels of MYC. Consistent with the low affinity of MYC:MAX for the NE *in vitro* (Fig 6), the switch from low to high MYC expression resulted in a notable increase in both the total number and average height of NE-containing summits (~7-fold and ~9-fold, respectively) (Fig 8E). In contrast, the number of peaks and average height of CME-containing summits were considerably less sensitive to MYC protein concentration (~2-fold and ~5-fold, respectively) (Fig 8E). Furthermore, the differential effects of MYC expression levels on the number of NE versus CME sites occupied were more pronounced at distal intergenic and intragenic sites compared to promoter regions (Fig 8F). Nonetheless, induction of high MYC levels enhanced the intensity of MYC binding to NE motifs within both promoters and distal regions. Examples of an increase in size of NE-containing peaks in high MYC-expressing cells are provided in Fig 8G (for the promoters of *SLC6A6* and *MTF2* genes) and in S6 Fig (for other promoter, intergenic, and intragenic regions). These results suggest that MYC overexpression is important for binding NE sites *in vivo*, especially at distal regions.

To assess whether high-affinity CME and low-affinity NE sequences mediate the same or distinct MYC functions, we performed gene ontology analyses of promoters with MYC-bound CME motifs (CME summits lacking the NE) or MYC-bound NE motifs (NE summits lacking the CME). As expected, MYC-bound CME promoter sites are found in genes involved in a variety of biological processes, including most prominently ribosome biogenesis and cellular metabolism. In contrast, genes with MYC-occupied NE promoter sequences are not generally involved in these top biological processes, but instead appear to have a preference for the DNA damage response (S7 Fig and S2 Table).

Importantly, we repeated all the above analyses of MYC-bound genomic sequences in a different human cell line (U2OS) by re-analyzing published ChIP-seq datasets obtained under low endogenous MYC levels and after doxycycline–induced MYC overexpression [28] and obtained similar results (S5 and S8 Figs, and S3 Table).

## Discussion

Using an unbiased PBM approach we have identified a set of 27 high-confidence hexamer motifs that are specifically bound by both MYC:MAX and MAX:MAX complexes *in vitro* (sequences in bold in Fig 1E). These include not only the CME sequence CACGTG and other E-boxes and E-box variants, but also non-E-box motifs such as the palindromic sequence AACGTT (referred to as NE), which was not previously known to be a specific MYC/MAX binding element. Notably, we verified by EMSA that both MYC:MAX and MAX:MAX complexes bound *in vitro* to the new AACGTT motif in a sequence-specific manner despite the ~20–30 fold lower affinity of MYC/MAX complexes for this sequence compared to the CME. The PBM results further indicated a contribution of the nucleotides immediately flanking the core 6-mer motifs, which supports previous observations made *in vitro* [5] and *in vivo* [17, 23]. In particular, the PBM indicated that CACGTGa (or its reverse complement tCACGTG) is disfavored by both MYC:MAX and MAX:MAX complexes, which is in accord with early site selection and EMSA experiments [5].

In addition, the PBM analyses identified low complexity G/C-rich sequences (AGGGGG, AGCGGG, AGGGGC, and CGGGGG) that were bound relatively strongly by MYC:MAX. However, these probes were not bound by MAX:MAX. This suggests that MYC may bind to these PBM probes in a sequence-independent manner that may not involve the basic region DNA-binding residues conserved between MYC and MAX. Although this observation is intriguing given the fact that *in vivo* MYC often binds genomic loci associated with CpG islands [17, 19, 20], the mode of recognition of these G/C-rich probes (sequence versus structural context recognition) needs to be further investigated by additional methods. Thus, our *in vitro* binding studies demonstrate that MYC/MAX complexes bind to a variety of non-canonical sequences and recognize low-affinity motifs, such as the non-E-box motif AACGTT, in a sequence-specific manner. The recognition of low-affinity non-E-box sequences is distinct from the non-specific DNA binding activity of MYC:MAX that was observed *in vitro* at high protein and DNA concentrations in the absence of non-specific competitor DNA [23].

The crystal structures of MYC:MAX and MAX:MAX complexes bound to DNA indicate that both MYC and MAX make identical contacts with the bases on either arm of the CME palindromic sequence [14, 15]. These include hydrogen bonding with the first and last base pairs of the CME (CACGTG), which are substituted in the NE motif (AACGTT). Molecular modeling of these substitutions in the MAX:MAX-DNA co-crystal structure [14] suggests a possible explanation as to why the NE motif is recognized by MYC/MAX complexes, albeit with a lower affinity. We propose that the first and last base pairs (AACGTT) of the NE may form a strong hydrogen bond (2.99 Å) between the thymine (T) and the His28 residue of MAX, while the complementary adenine (A) makes only a weak van der Waals contact with Glu32 of MAX (4.26Å). Other base pairs at these positions (distinct from CME or NE) would either clash with the structure or be too distant to make contact with MAX (see S9 Fig).

Analysis of chromatin loci bound by MYC in the genome of human P493-6 B-cells indicated that ~87% of all MYC-occupied sequences (ChIP-seq summits) contain at least one of the top 21 MYC:MAX-bound motifs identified by the PBM (excluding the low complexity G/C-rich motifs, Fig 1E) in both low- and high-MYC expressing cells (S10 Fig). Notably, we found that the NE motif AACGTT is present within MYC-bound genomic sequences (in both P493-6 and U2OS cells) at frequencies comparable to those of many E-box motifs. These observations support the previous finding that MYC often binds genomic loci that lack high affinity E-boxes [23]. However, that does not mean that such binding is necessarily “non-specific”, as previously suggested [23]. Rather, we propose that MYC binding to low-affinity sites such as AACGTT may contribute to cooperative DNA binding with other transcription factors or chromatin-associated factors, in accord with recent findings [21, 23, 25, 28, 40].

In human cells (P493-6 and U2OS) MYC bound both the CME (CACGTG) and the AACGTT motif preferentially within promoter regions and MYC overexpression increased binding to both types of promoter motifs. Notably, however, the most prevalent functions of genes with MYC-bound CME promoter motifs (ribosome biogenesis and metabolic processes) were not enriched in the genes with MYC-occupied AACGTT promoter motifs, which tended to be involved in the DNA damage response. Moreover, MYC overexpression preferentially increased the number of occupied AACGTT sites relative to CME sites, especially at distal inter/intragenic loci (Fig 8F and S8 Fig). Hence, MYC expression levels and recognition of distinct DNA sequences with different affinities might influence distinct genetic programs, as was recently proposed [28].

In conclusion, our results suggest that overexpression of MYC allows for sequence-specific binding to the NE motif AACGTT, and raise the intriguing possibility that the marked induction of *de novo* MYC binding to low-affinity AACGTT-containing genomic loci (especially those distal to promoters) could be important for the oncogenic effects of MYC overexpression that is characteristic of many cancers.

## Supporting information

S1 Fig Effects of left-flanking 3-mer sequences on binding of MAX:MAX and MYC:MAX to the core CME. CME-containing PBM probes with all possible left-flanking 3-mers were ranked according to their binding to MYC:MAX and MAX:MAX. Logos of position weight matrices were obtained with MEME for the top 25%, 25–50%, 50–75%, and 75–100% of bound probes.
(PDF)

S2 Fig Effects of right-flanking 3-mer sequences on binding of MAX:MAX and MYC:MAX to the core CME. CME-containing PBM probes with all possible right-flanking 3-mers were ranked according to their binding to MYC:MAX and MAX:MAX. Logos of position weight matrices were obtained with MEME for the top 25%, 25–50%, 50–75%, and 75–100% of bound probes.
(PDF)

S3 Fig Effects of flanking 3-mer sequences on binding of MAX:MAX versus MYC:MAX. Scatter plots of MYC:MAX and MAX:MAX binding scores for CME-containing PBM probes with all possible left-flanking (top) and right-flanking (bottom) 3-mers are shown. Probes in red indicate those with trimer sequences that may favor binding by one of the complexes. Underlined are favored left-flanking nucleotides supported by the position weigh matrices of S1 Fig.
(PDF)

S4 Fig Effects of mutations in tgaCACGTGgta on binding of MAX:MAX versus MYC:MAX. Scatter plots show MYC:MAX and MAX:MAX binding scores for PBM probes containing random 3–6 substitutions in 12-mer tgaCACGTGgta (probe shown in black). Top plot shows all probes with binding score above 80% random threshold and the double dashed line indicates the 95% binding threshold. Bottom shows only probes above 95% random threshold. Red indicates probes with mutations that preferentially decrease binding to either MYC:MAX or MAX:MAX. Mutations are underlined.
(PDF)

S5 Fig Frequency and distribution of motifs within MYC ChIP-seq peaks in two human cell lines overexpressing MYC. **(A)** Comparison of the MYC/MAX affinity for different motifs (verified by EMSA) with the normalized frequency of motifs under MYC ChIP-seq summits (-100/+100 bp) in the genome of P493-6 and U2OS cells overexpressing MYC. The in vitro affinities High, Medium, Low and non-detectable (n.d.) relate to Fig 6. **(B)** Comparison of motif frequency distribution within CME and NE summits (-100/+100 bp) versus their flanking 500bp regions (summits extended +/- 250 bp on each flank). **(C)** Frequency distribution of the indicated motifs within their MYC summits (-100/+100 bp). Only the CME shows a reproducible clustering at the center of the summit region in both cell lines. Motif frequency distributions were obtained with Homer peak annotation tool.
(PDF)

S6 Fig Examples of MYC ChIP-seq peaks in the genome of human P493-6 cells under low and high MYC expression conditions. Tracks are from Genome Browser. Relates to Fig 8G.
(PDF)

S7 Fig Gene ontology analyses of promoters with MYC-associated CME or NE motifs. Enrichment of biological processes associated with gene promoters having MYC-bound CME **(A)** or NE **(B)** motifs (ChIP-seq summits) in MYC-overexpressing P493-6 cells were obtained with Metascape and are ranked by significance (-log10 P value). See also S2 Table for the lists of genes.
(PDF)

S8 Fig Characterization of MYC-occupied CME (CACGTG) and NE (AACGTT) genomic loci and influence of MYC expression levels in U2OS cells. **(A)** MYC-bound sequences were obtained from published ChIP-seq datasets of U2OS cells expressing low endogenous MYC (low-MYC) or overexpressing MYC (High-MYC) [28]. The frequencies of all ten possible E-boxes (CANNTG), the NE motif AACGTT, and two control (Ctrl) sequences under MYC ChIP-seq summits were normalized to the occurrence of each motif in the human genome. The summits are the ±100 bp region centered at the apex of ChIP-seq peaks. **(B)** Venn diagram shows the number of MYC ChIP-seq summits that contain the CME, the NE or both motifs in high MYC-expressing U2OS cells. **(C)** Frequency distribution plots show the fraction (%) of all MYC ChIP-seq summits and those specifically containing the CME or NE motifs that are located within promoters (± 2 kb from a TSS), intergenic or intragenic regions. **(D)** Effect of MYC overexpression on the number and average (mean) height of MYC ChIP-seq summits is shown as fold change (high MYC vs. low MYC) for the summits containing the CME or NE motifs. **(E)** Effect of MYC overexpression on the number of MYC ChIP-seq summits at promoters, intergenic and intragenic regions is shown as fold change (high MYC vs. low MYC) for the specific CME- or NE-containing summits, and for all summits. **(F)** Gene ontology analyses of promoters with MYC-associated CME (top) or NE (bottom) summits in MYC-overexpressing U2OS cells. Enrichment of biological processes was obtained with Metascape and processes are ranked by significance (-log10 P value). See also S3 Table for the lists of genes associated with each biological process.
(PDF)

S9 Fig Diagram illustrating a possible mechanism for sequence-specific recognition of the NE motif by MYC/MAX complexes. **(Left)** The contacts between MAX:MAX and the CME motif are depicted based on published X-ray crystallographic results [14] (PDBe 1an2). Arrows indicate H-bonds and the corresponding distance between base and amino acid residues is indicated. Only the contacts by one MAX monomer with on one half of the symmetric palindrome are shown. **(Right)** Proposed interactions of MAX with the NE motif were modeled by using the Coot (Crystallographic Object-Oriented Toolkit) software. The postulated new hydrogen bond between His28 (H28) of MAX and O4 of Thymine (T) is indicated with a red arrow. A potential van der Waals contact is indicated with a dashed red arrow.
(PDF)

S10 Fig Most MYC-bound genomic sites in P493-6 cells contain at least one of the top 21 MYC:MAX-bound motifs identified by the PBM. **(A)** Total number of MYC ChIP-seq summits (all summits) and summits having one of the top 21 motifs for MYC:MAX identified in vitro by the PBM (i.e., MYC:MAX motifs of Fig 1E excluding GC-rich motifs in italics) under low and high MYC expression. **(B)** Fraction (%) of MYC summits with one of the top 21 MYC:MAX-bound motifs identified *in vitro*.
(PDF)

S1 Table PBM probes sequences and MYC/MAX binding scores. (XLSX)

S2 Table Gene ontology of MYC-bound CME and NE sites in P493-6 cells. (XLSX)

S3 Table Gene ontology of MYC-bound C’ME and NE sites in U2OS cells. (XLSX)
