# Supplementary figures and images for: Sequence-specific DNA binding by MYC/MAX to low-affinity non-E-box motifs
Source: PLoS One. 2017 Jul 18;12(7):e0180147. doi: 10.1371/journal.pone.0180147 (PMC5515408; doi:10.1371/journal.pone.0180147)

# MAX:MAX

# MYC:MAX

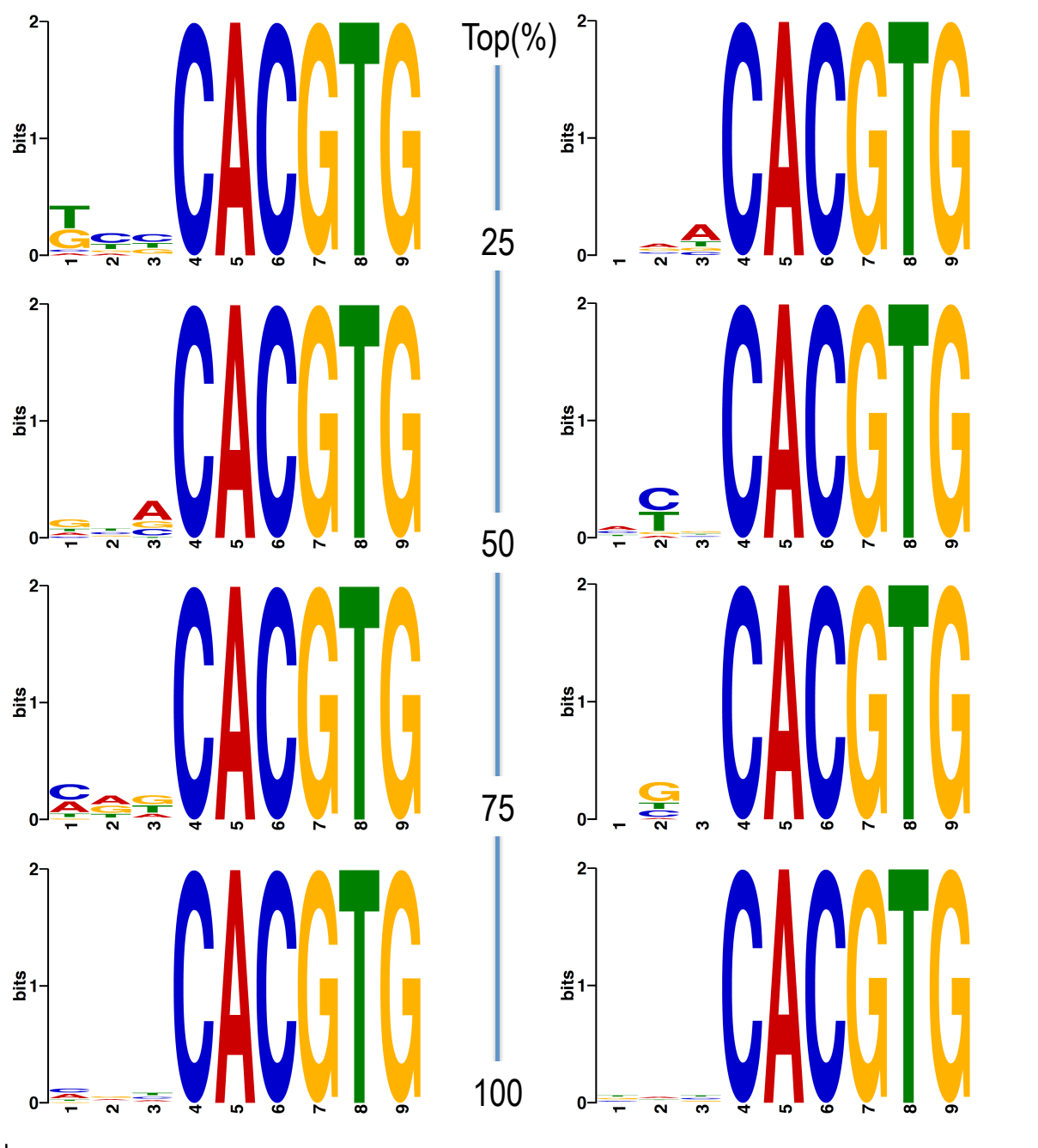

nnnCACGTGgta

Supplement: S1 Fig — CME-containing PBM probes with all possible left-flanking 3-mers were ranked according to their binding to MYC:MAX and MAX:MAX. Logos of position weight matrices were obtained with MEME for the top 25%, 25–50%, 50–75%, and 75–100% of bound probes. (PDF) [file pone.0180147.s001.pdf]

MAX:MAX

MYC:MAX

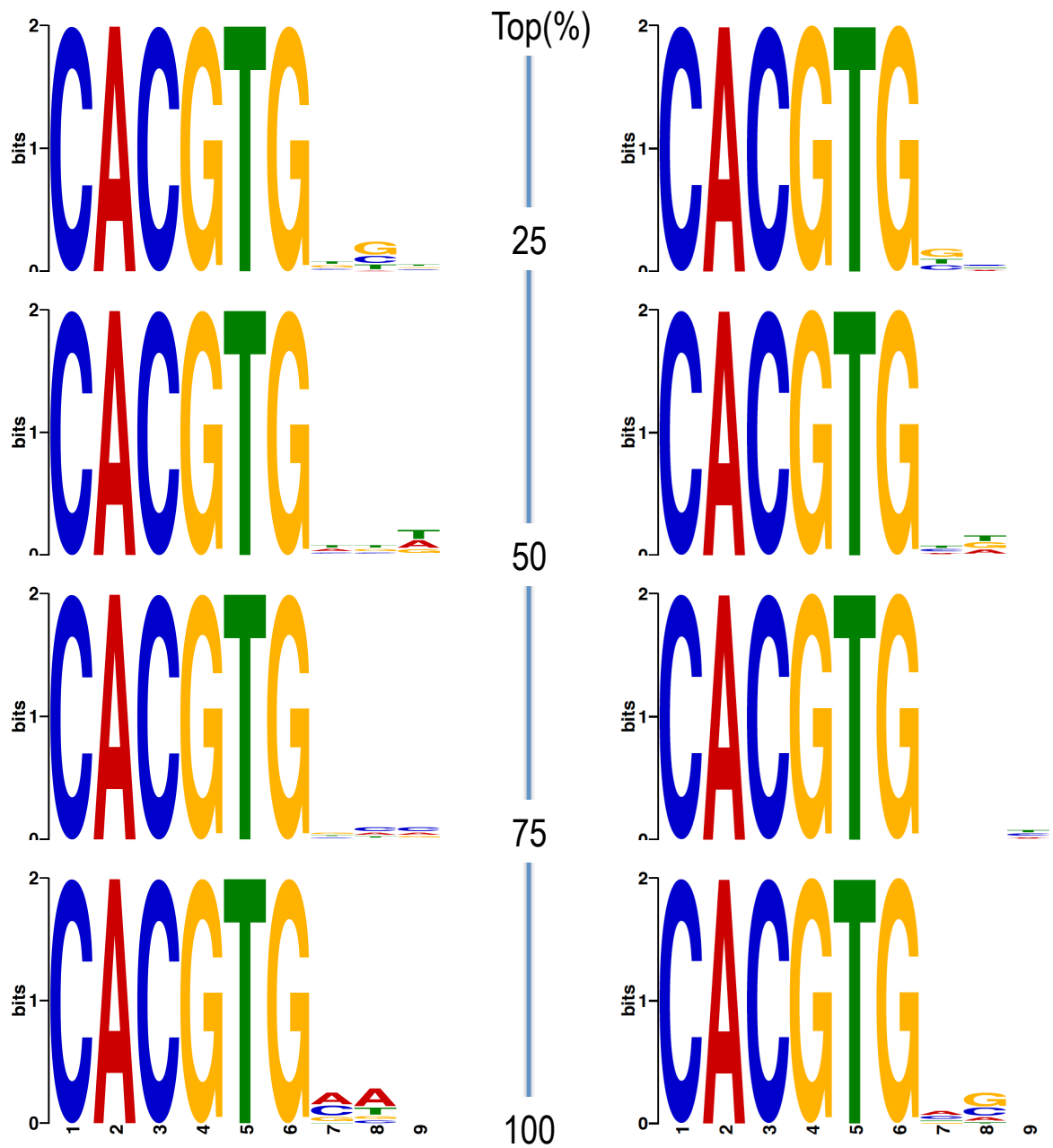

tgaCACGTGnnn

Supplement: S2 Fig — CME-containing PBM probes with all possible right-flanking 3-mers were ranked according to their binding to MYC:MAX and MAX:MAX. Logos of position weight matrices were obtained with MEME for the top 25%, 25–50%, 50–75%, and 75–100% of bound probes. (PDF) [file pone.0180147.s002.pdf]

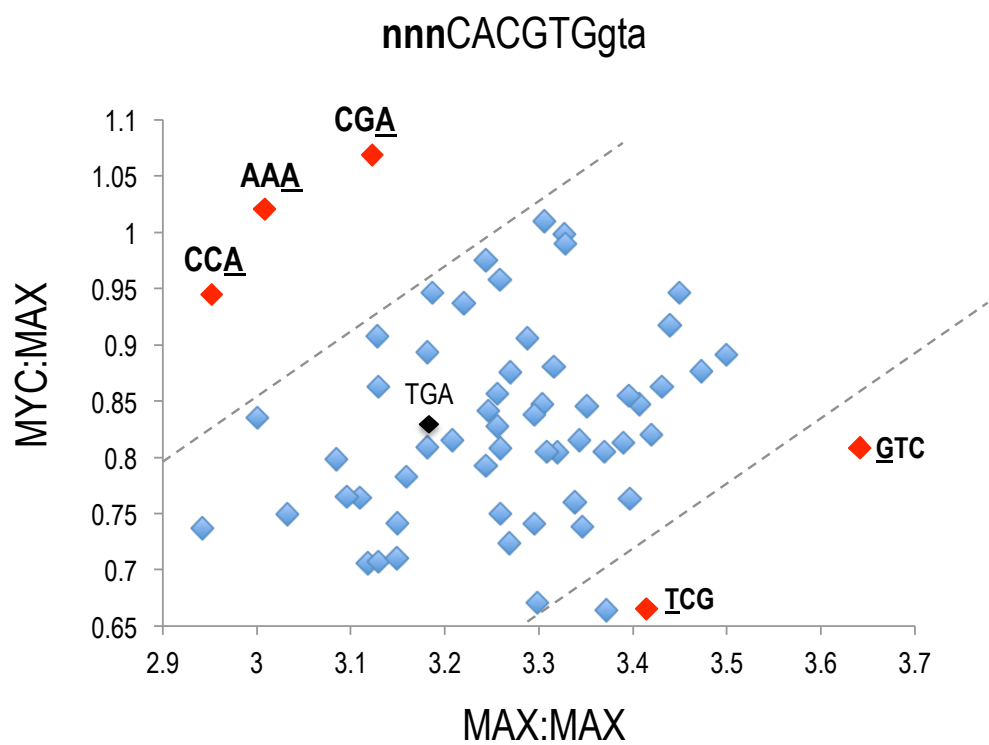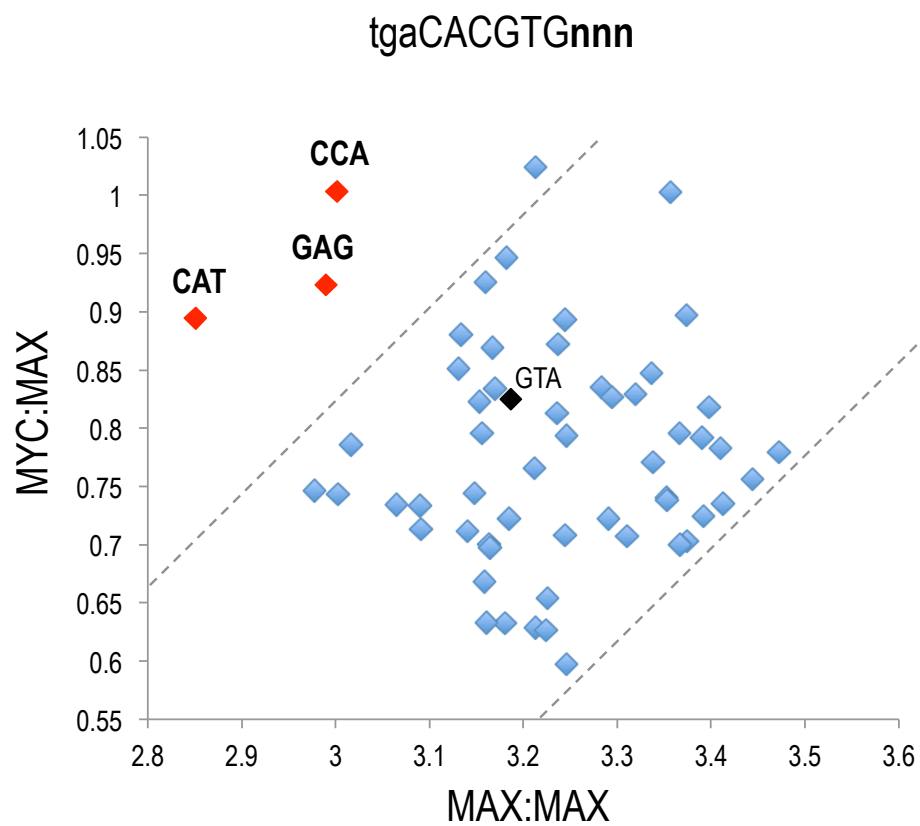

Supplement: S3 Fig — Scatter plots of MYC:MAX and MAX:MAX binding scores for CME-containing PBM probes with all possible left-flanking (top) and right-flanking (bottom) 3-mers are shown. Probes in red indicate those with trimer sequences that may favor binding by one of the complexes. Underlined are favored left-flanking nucleotides supported by the position weigh matrices of S1 Fig. (PDF) [file pone.0180147.s003.pdf]

# Effects of mutations in 12-mer tgaCACGTGgta

PBM scores >80% random probes

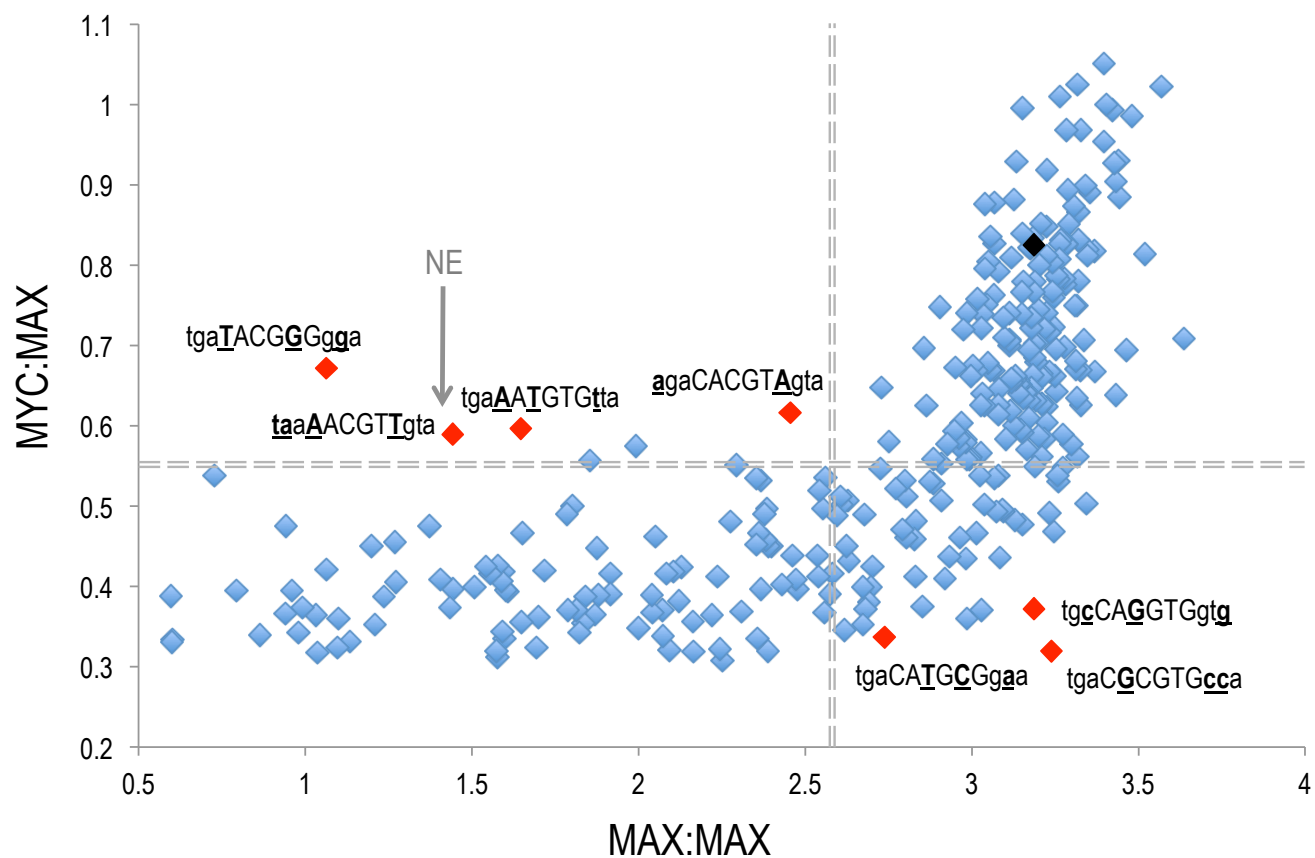

PBM scores >95% random probes

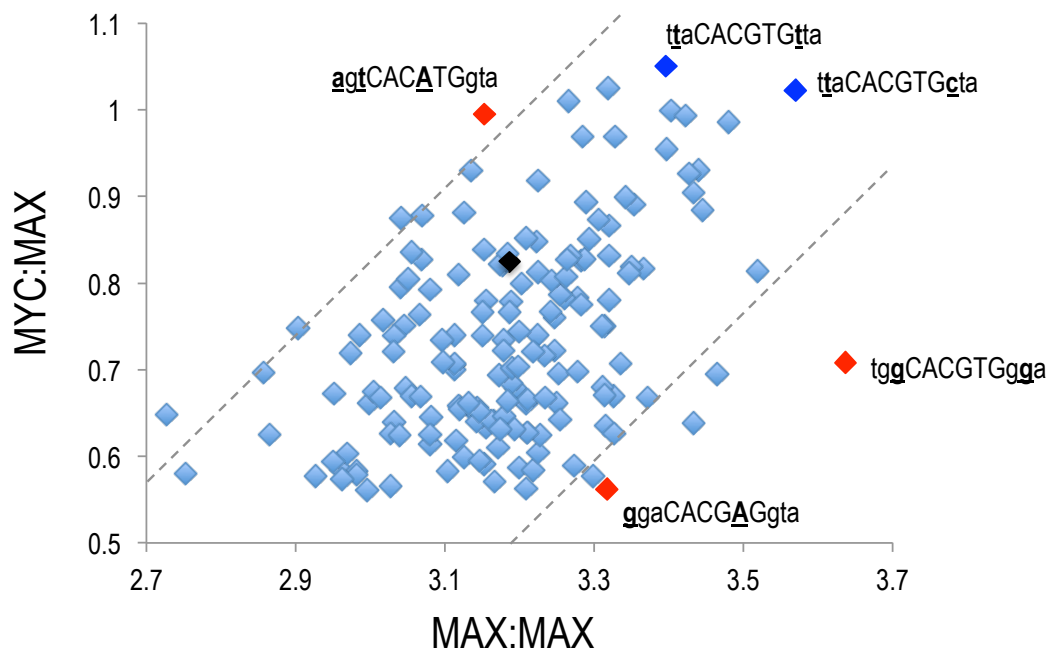

Supplement: S4 Fig — Scatter plots show MYC:MAX and MAX:MAX binding scores for PBM probes containing random 3–6 substitutions in 12-mer tgaCACGTGgta (probe shown in black). Top plot shows all probes with binding score above 80% random threshold and the double dashed line indicates the 95% binding threshold. Bottom shows only probes above 95% random threshold. Red indicates probes with mutations that preferentially decrease binding to either MYC:MAX or MAX:MAX. Mutations are underlined. (PDF) [file pone.0180147.s004.pdf]

**A**

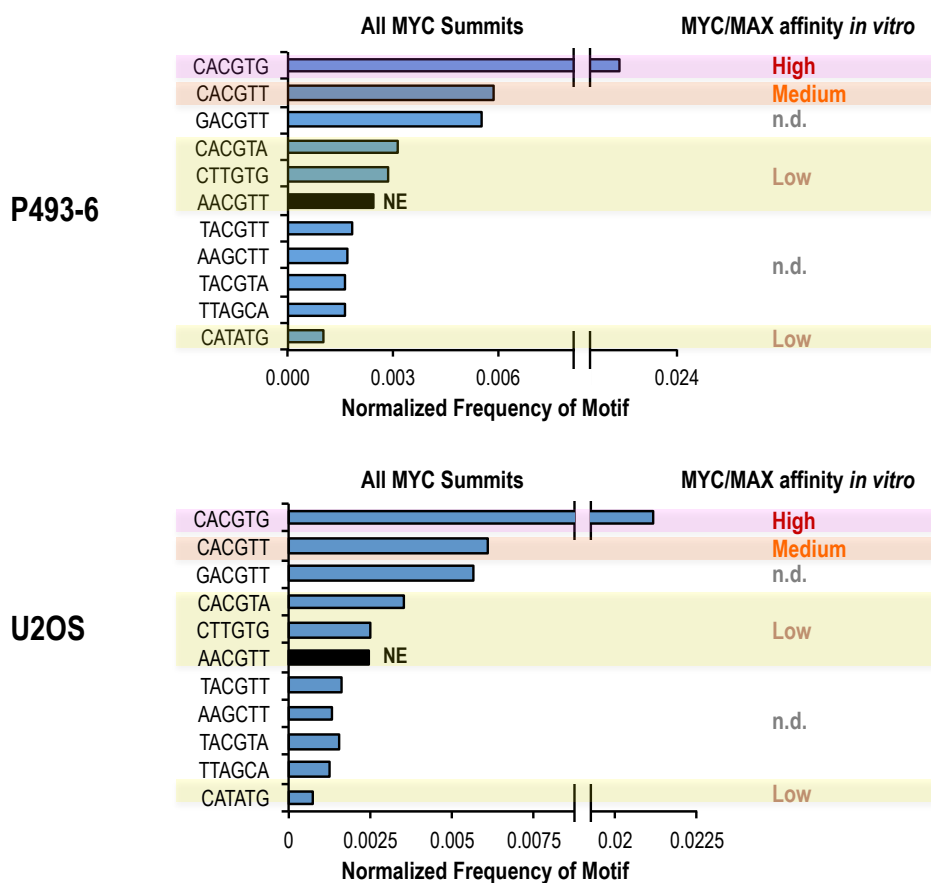

**B**

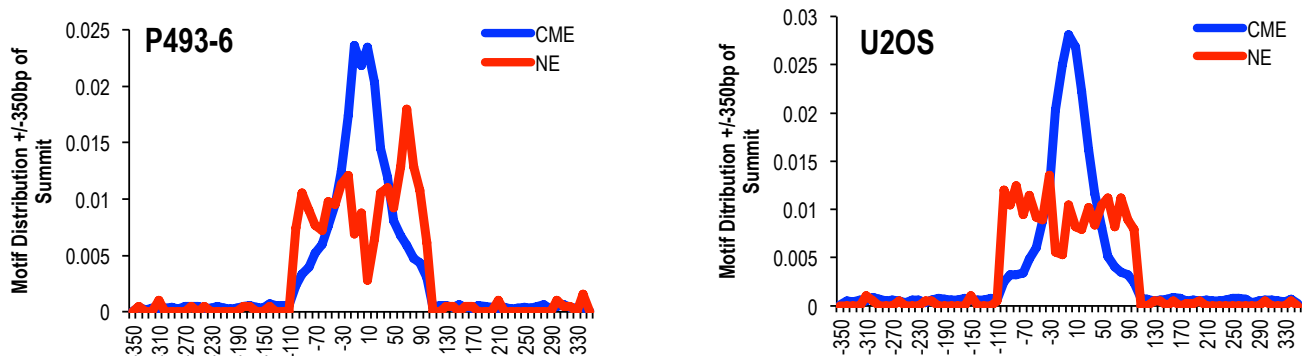

**C**

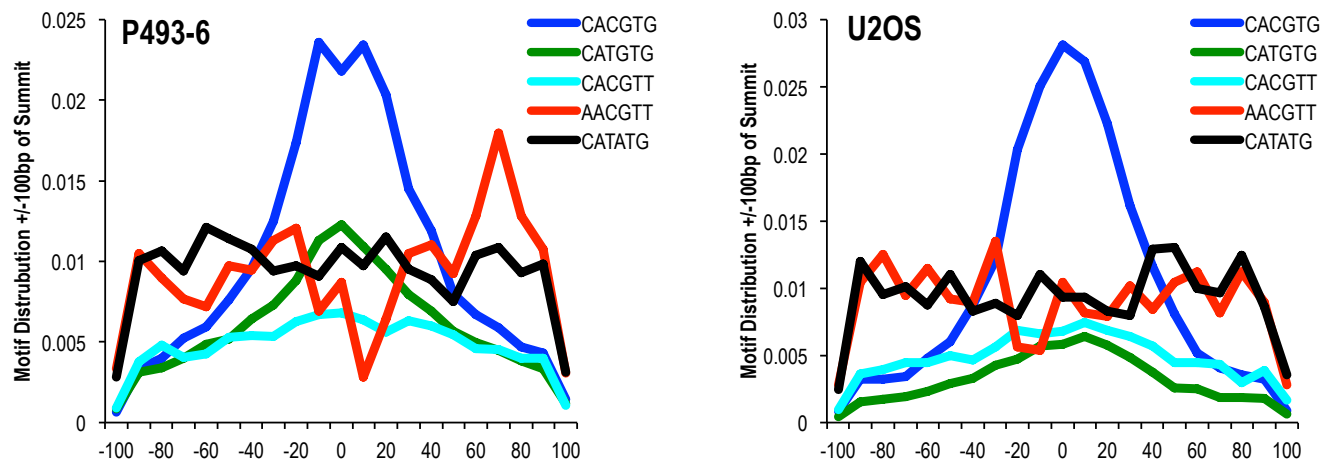

Supplement: S5 Fig — (A) Comparison of the MYC/MAX affinity for different motifs (verified by EMSA) with the normalized frequency of motifs under MYC ChIP-seq summits (-100/+100 bp) in the genome of P493-6 and U2OS cells overexpressing MYC. The in vitro affinities High, Medium, Low and non-detectable (n.d.) relate to Fig 6. (B) Comparison of motif frequency distribution within CME and NE summits (-100/+100 bp) versus their flanking 500bp regions (summits extended +/- 250 bp on each flank). (C) Frequency distribution of the indicated motifs within their MYC summits (-100/+100 bp). Only the CME shows a reproducible clustering at the center of the summit region in both cell lines. Motif frequency distributions were obtained with Homer peak annotation tool. (PDF) [file pone.0180147.s005.pdf]

# Promoter +/-2kB

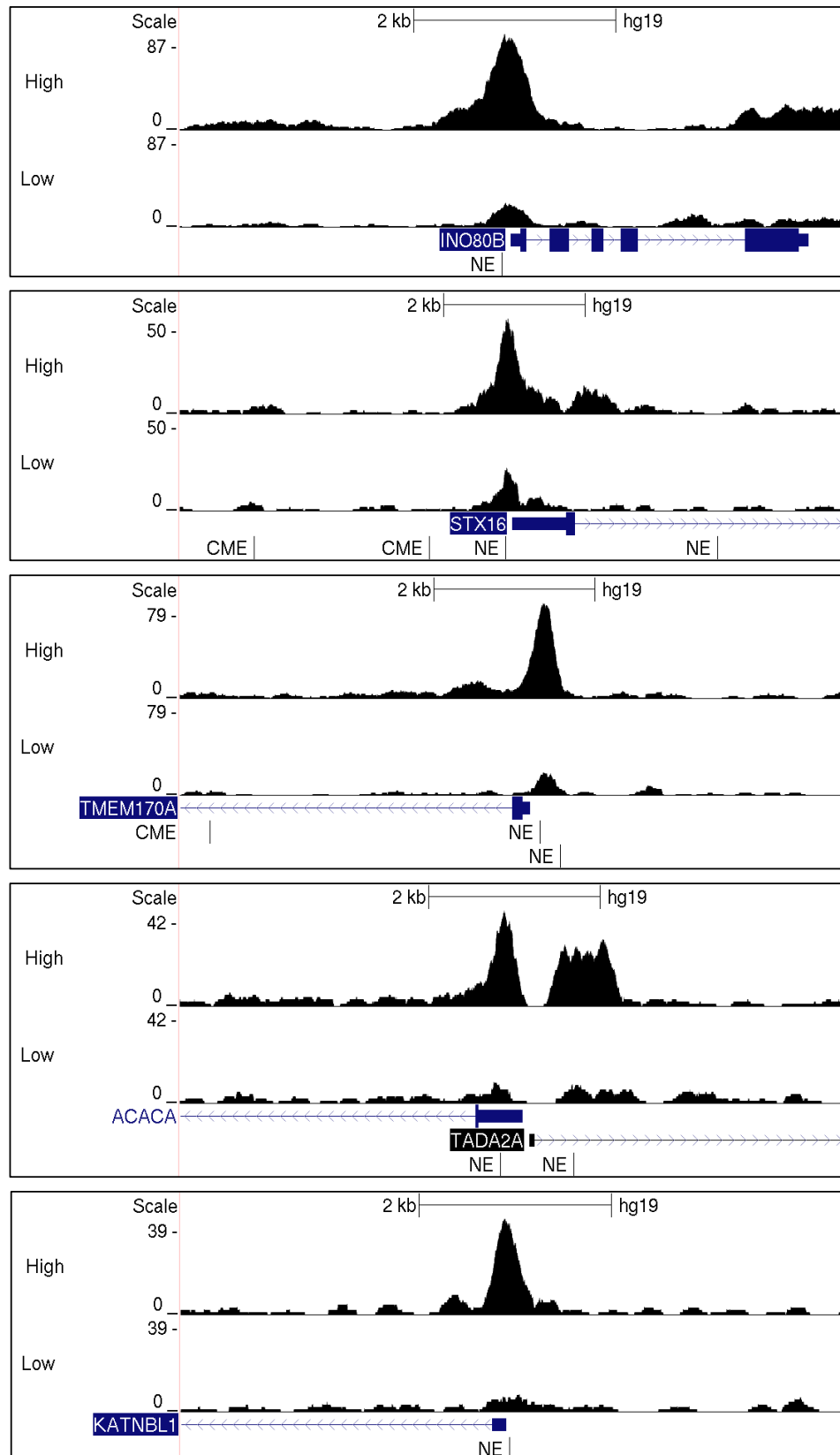

# Promoter +/-2kB

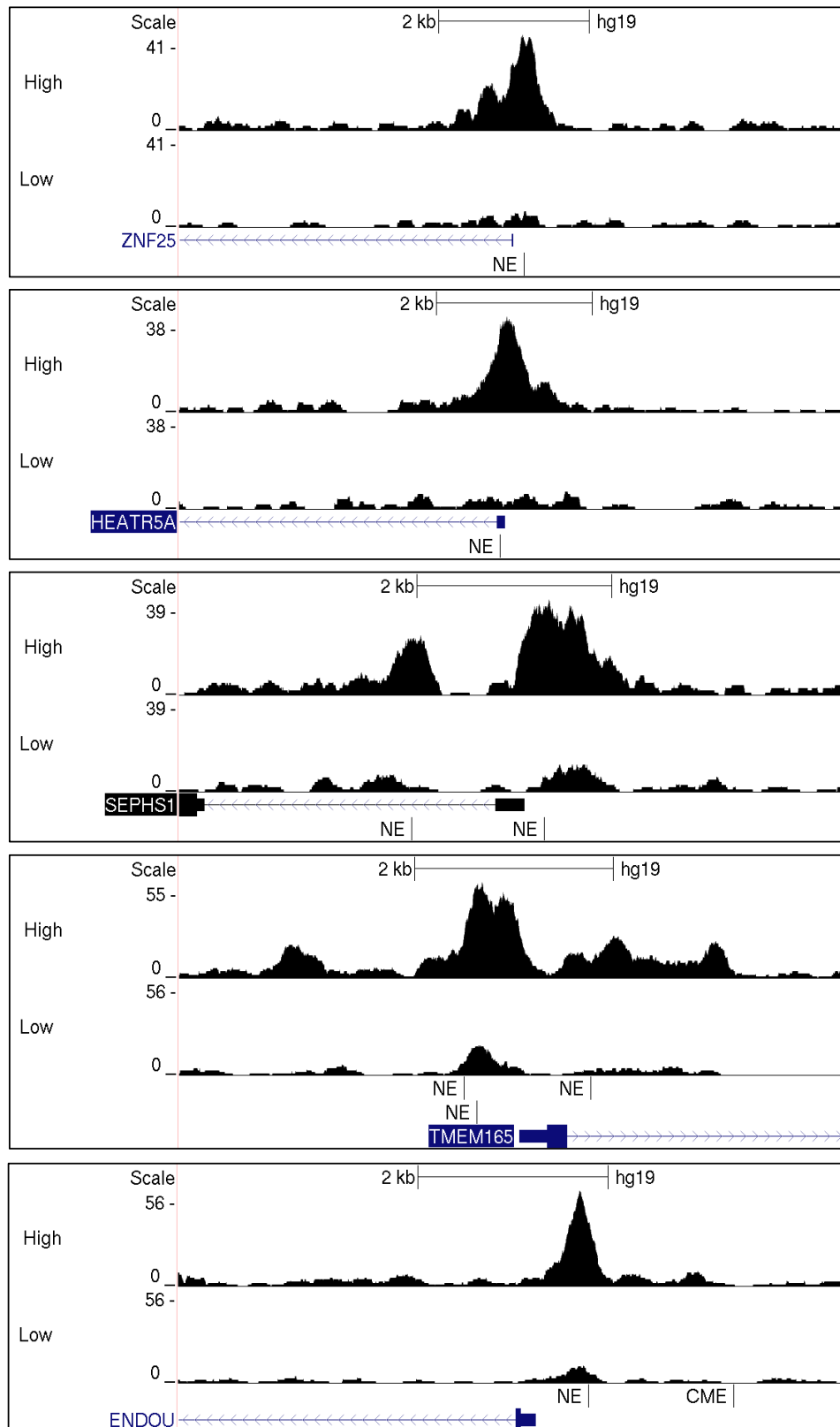

# Intergenic

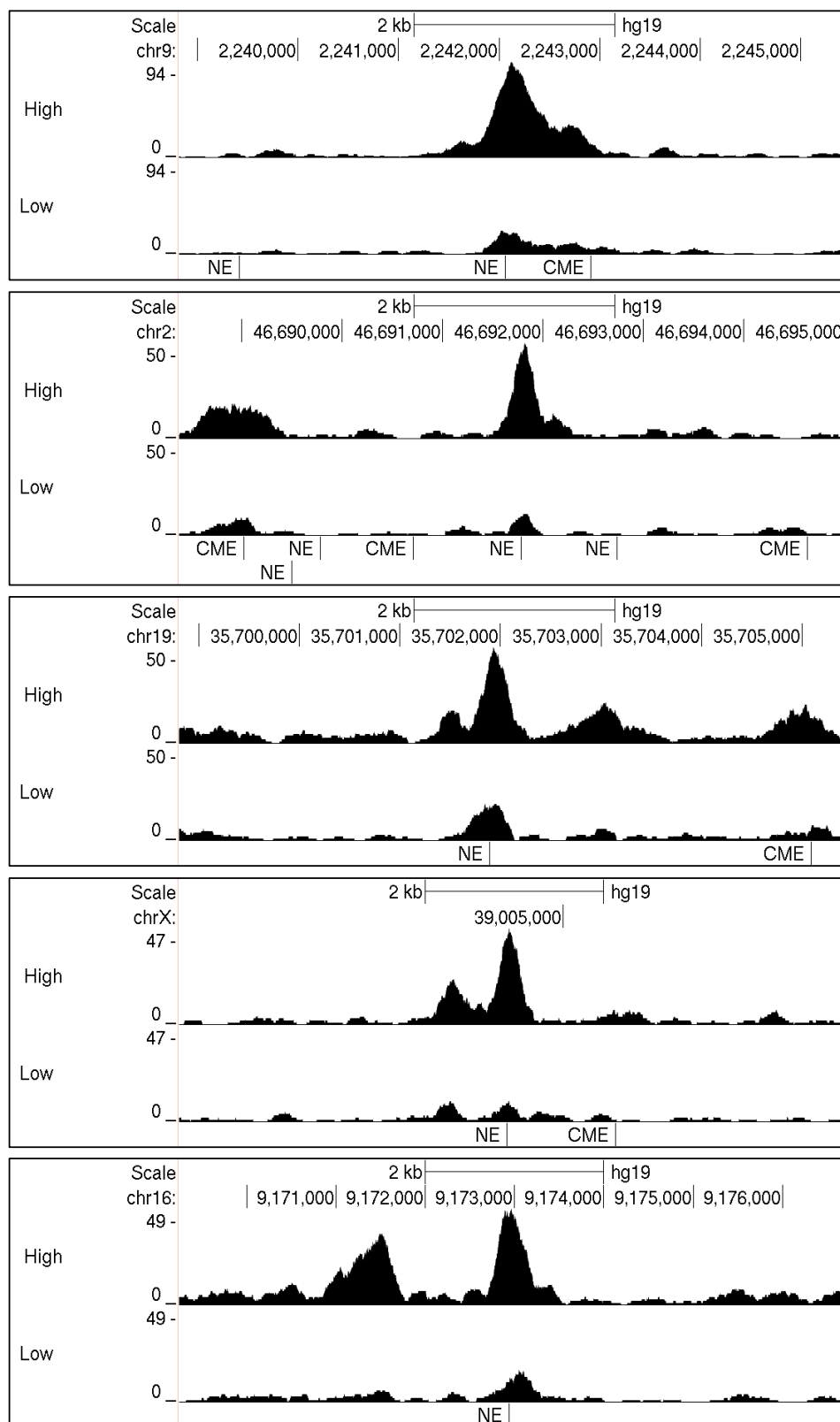

# Intragenic

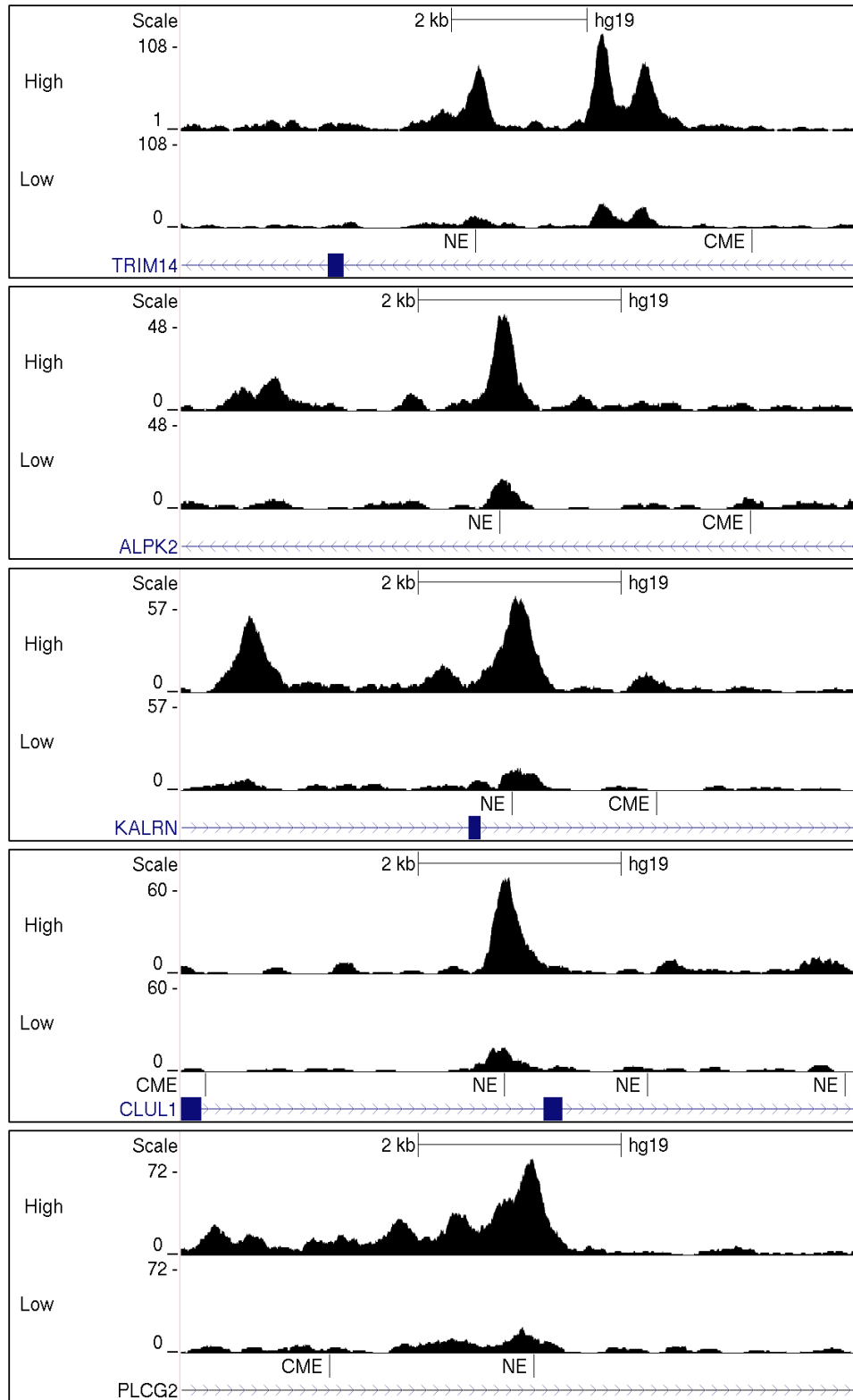

Supplement: S6 Fig — Tracks are from Genome Browser. Relates to Fig 8G. (PDF) [file pone.0180147.s006.pdf]

**A** MYC summits containing only the CME motif

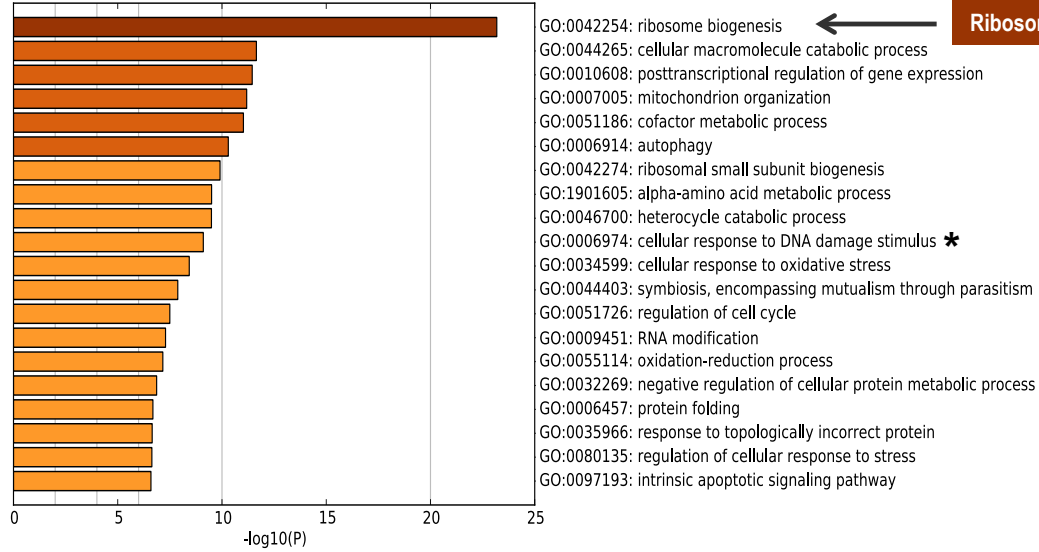

**B** MYC summits containing only the NE motif

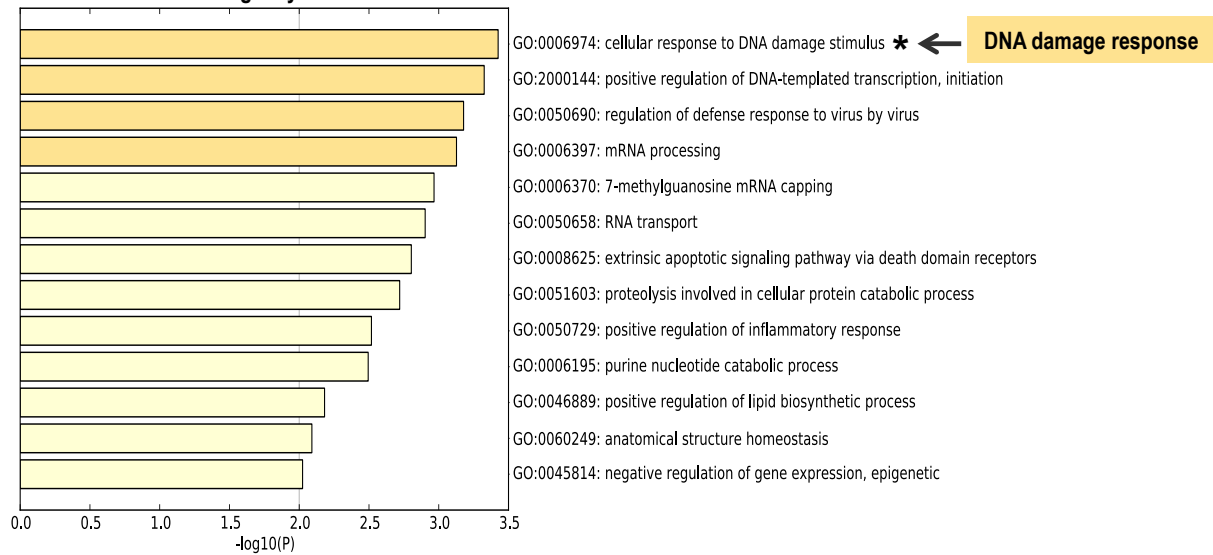

Supplement: S7 Fig — Enrichment of biological processes associated with gene promoters having MYC-bound CME (A) or NE (B) motifs (ChIP-seq summits) in MYC-overexpressing P493-6 cells were obtained with Metascape and are ranked by significance (-log10 P value). See also S2 Table for the lists of genes. (PDF) [file pone.0180147.s007.pdf]

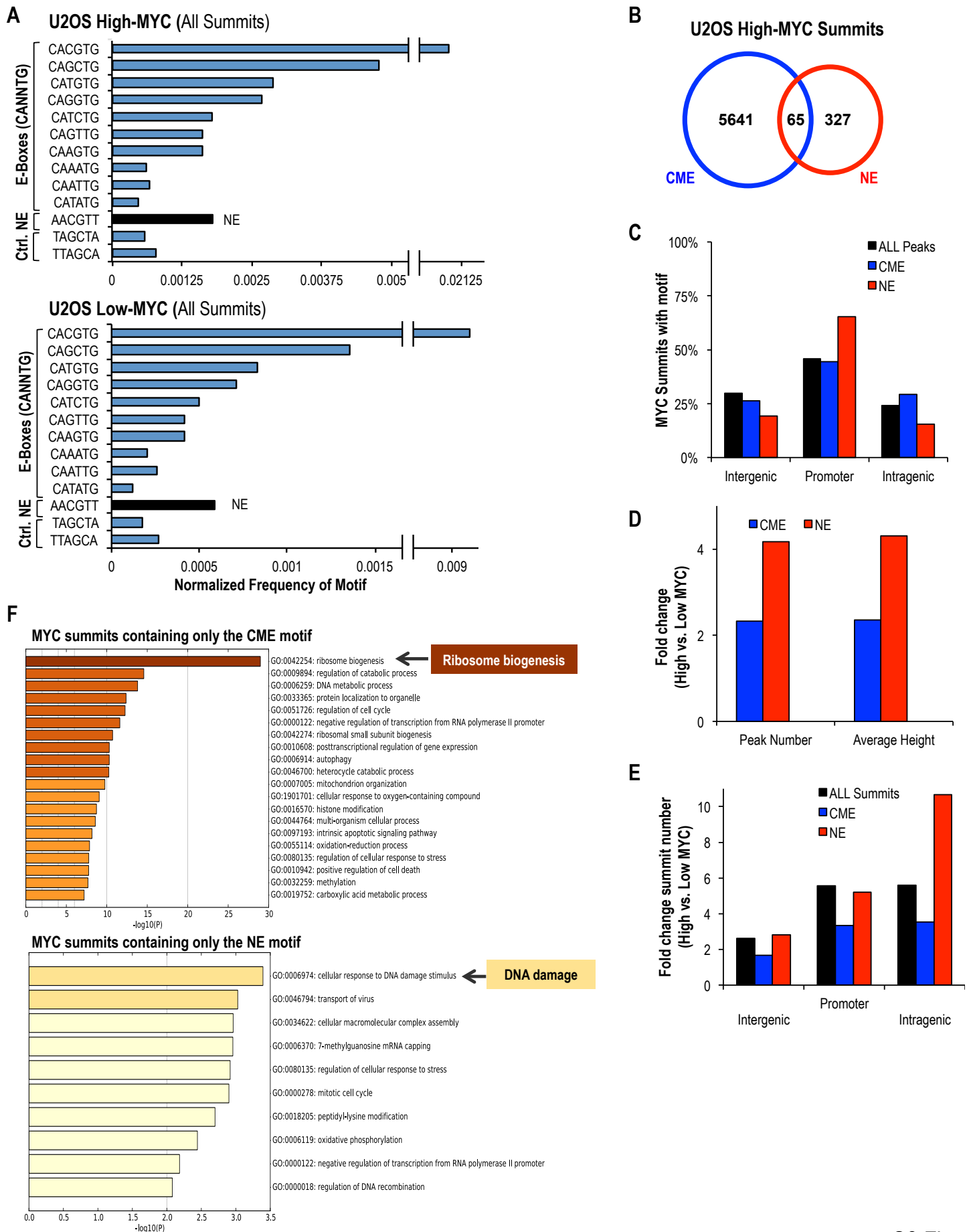

Supplement: S8 Fig — (A) MYC-bound sequences were obtained from published ChIP-seq datasets of U2OS cells expressing low endogenous MYC (low-MYC) or overexpressing MYC (High-MYC) [28]. The frequencies of all ten possible E-boxes (CANNTG), the NE motif AACGTT, and two control (Ctrl) sequences under MYC ChIP-seq summits were normalized to the occurrence of each motif in the human genome. The summits are the ±100 bp region centered at the apex of ChIP-seq peaks. (B) Venn diagram shows the number of MYC ChIP-seq summits that contain the CME, the NE or both motifs in high MYC-expressing U2OS cells. (C) Frequency distribution plots show the fraction (%) of all MYC ChIP-seq summits and those specifically containing the CME or NE motifs that are located within promoters (± 2 kb from a TSS), intergenic or intragenic regions. (D) Effect of MYC overexpression on the number and average (mean) height of MYC ChIP-seq summits is shown as fold change (high MYC vs. low MYC) for the summits containing the CME or NE motifs. (E) Effect of MYC overexpression on the number of MYC ChIP-seq summits at promoters, intergenic and intragenic regions is shown as fold change (high MYC vs. low MYC) for the specific CME- or NE-containing summits, and for all summits. (F) Gene ontology analyses of promoters with MYC-associated CME (top) or NE (bottom) summits in MYC-overexpressing U2OS cells. Enrichment of biological processes was obtained with Metascape and processes are ranked by significance (-log10 P value). See also S3 Table for the lists of genes associated with each biological process. (PDF) [file pone.0180147.s008.pdf]

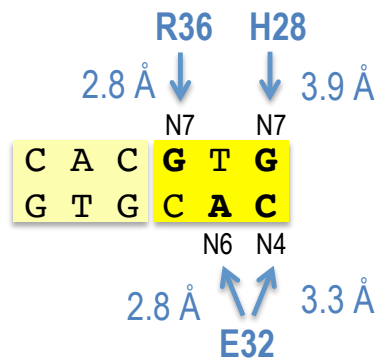

CME

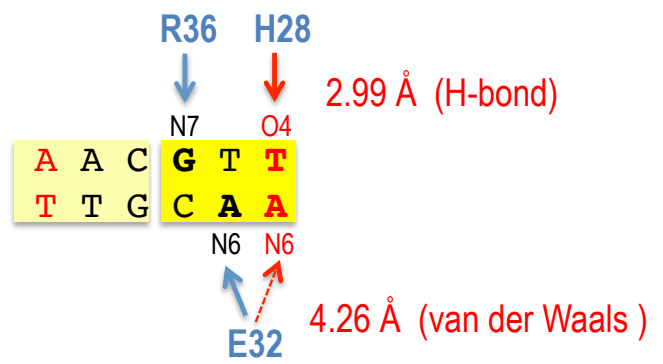

NE

Supplement: S9 Fig — (Left) The contacts between MAX:MAX and the CME motif are depicted based on published X-ray crystallographic results [14] (PDBe 1an2). Arrows indicate H-bonds and the corresponding distance between base and amino acid residues is indicated. Only the contacts by one MAX monomer with on one half of the symmetric palindrome are shown. (Right) Proposed interactions of MAX with the NE motif were modeled by using the Coot (Crystallographic Object-Oriented Toolkit) software. The postulated new hydrogen bond between His28 (H28) of MAX and O4 of Thymine (T) is indicated with a red arrow. A potential van der Waals contact is indicated with a dashed red arrow. (PDF) [file pone.0180147.s009.pdf]

**A**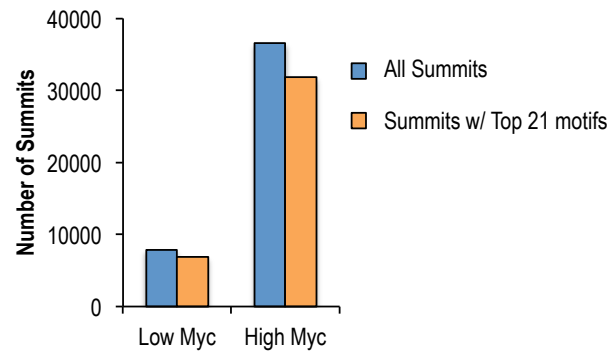**B**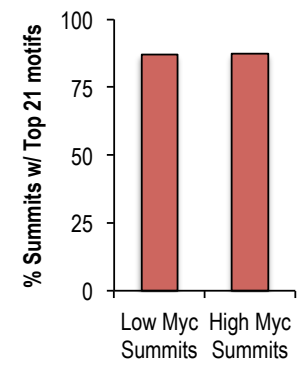

Supplement: S10 Fig — (A) Total number of MYC ChIP-seq summits (all summits) and summits having one of the top 21 motifs for MYC:MAX identified in vitro by the PBM (i.e., MYC:MAX motifs of Fig 1E excluding GC-rich motifs in italics) under low and high MYC expression. (B) Fraction (%) of MYC summits with one of the top 21 MYC:MAX-bound motifs identified in vitro. (PDF) [file pone.0180147.s010.pdf]
